# The Predicted Mannosyltransferase GT69-2 Antagonizes RFW-1 To Regulate Cell Fusion in Neurospora crassa

**DOI:** 10.1128/mBio.00307-21

**Published:** 2021-03-16

**Authors:** Yang Li, Jens Heller, A. Pedro Gonçalves, N. Louise Glass

**Affiliations:** a Department of Plant and Microbial Biology, University of California, Berkeley, California, USA; b Environmental Genomics and Systems Biology Division, Lawrence Berkeley National Laboratory, Berkeley, California, USA; University of Toronto

**Keywords:** *Neurospora*, cell fusion, allorecognition, mannosyltransferase, cell wall, chemotropism, glycosyltransferase, CAP59

## Abstract

Filamentous fungi undergo somatic cell fusion to create a syncytial, interconnected hyphal network which confers a fitness benefit during colony establishment. However, barriers to somatic cell fusion between genetically different cells have evolved that reduce invasion by parasites or exploitation by maladapted genetic entities (cheaters). Here, we identified a predicted mannosyltransferase, glycosyltransferase family 69 protein (GT69-2) that was required for somatic cell fusion in Neurospora crassa. Cells lacking GT69-2 prematurely ceased chemotropic signaling and failed to complete cell wall dissolution and membrane merger in pairings with wild-type cells or between Δ*gt69-2* cells (self fusion). However, loss-of-function mutations in the linked *regulator of cell fusion and cell wall remodeling-1* (*rfw-1*) locus suppressed the self-cell-fusion defects of Δ*gt69-2* cells, although Δ*gt69-2* Δ*rfw-1* double mutants still failed to undergo fusion with wild-type cells. Both GT69-2 and RFW-1 localized to the Golgi apparatus. Genetic analyses indicated that RFW-1 negatively regulates cell wall remodeling-dependent processes, including cell wall dissolution during cell fusion, separation of conidia during asexual sporulation, and conidial germination. GT69-2 acts as an antagonizer to relieve or prevent negative functions on cell fusion by RFW-1. In *Neurospora* species and N. crassa populations, alleles of *gt69-2* were highly polymorphic and fell into two discrete haplogroups. In all isolates within haplogroup I, *rfw-1* was conserved and linked to *gt69-2*. All isolates within haplogroup II lacked *rfw-1.* These data indicated that *gt69-2/rfw-1* are under balancing selection and provide new mechanisms regulating cell wall remodeling during cell fusion and conidial separation.

## Abstract (precis)

Cell wall remodeling is a dynamic process that balances cell wall integrity versus cell wall dissolution. In filamentous fungi, cell wall dissolution is required for somatic cell fusion and conidial separation during asexual sporulation.

## INTRODUCTION

In filamentous fungi, the interconnected mycelial network formed as a result of somatic cell fusion within an individual colony allows cytoplasm, nuclei, organelles, and nutrients to be shared, enhancing hyphal growth and rapid spatial expansion (1–5). Somatic cell fusion can occur between genetically identical and genetically dissimilar fungal cells and colonies. Fusion between genetically dissimilar cells/colonies can facilitate the introduction and maintenance of genetic variation in populations for adaptive processes (6, 7). In some pathogenic fungi, intra- or interfungal species cell fusion events are important for virulence and host colonization (8, 9), or are required to broaden host specificity (10). However, cell fusion between genetically nonidentical colonies or cells can result in the transfer of infectious elements, such as mycoviruses or selfish genetic elements, or colonization by debilitated genotypes, such as dysfunctional mitochondria (11–13). To avoid such exploitation, filamentous fungi have evolved a variety of mechanisms to govern non-self-recognition processes (allorecognition) during both pre- and post-cell-fusion events (13–17).

In the filamentous ascomycete species Neurospora crassa, cell-to-cell communication and chemotropic interactions have been extensively studied and are important aspects that occur prior to cell fusion (18). In genetically identical germlings, intercellular communication promotes the formation of specialized structures in germinated asexual spores (germlings) termed conidial anastomosis tubes (CATs) that undergo chemotropic growth (19). An essential part of chemotropic growth between germlings and hyphae is the oscillation of a MAK-2 MAP kinase signaling complex and the SOFT protein to opposing CAT tips (20–22). So far, approximately 80 genes have been identified that are involved in the process of communication and/or fusion, ranging in function from intracellular signaling, calcium modulation, membrane merger, production of reactive oxygen species, actin regulation, vesicle trafficking, and transcriptional control (18, 23, 24).

Recently, allorecognition between genetically different germlings was investigated using a population sample of N. crassa (25–29). Three key checkpoints were characterized that regulate allorecognition in germlings/hyphae during the cell fusion process (17). The first checkpoint is controlled by allelic specificity at the determinant of communication (*doc*) loci, where nonidentity negatively regulates chemotropic interactions (25). The second checkpoint blocks the transition from cell adhesion to cell wall dissolution when adhered cells have nonidentity of *cwr-1* and *cwr-2* (cell wall remodeling) loci (29). The third checkpoint occurs postfusion and triggers a rapid cell death response in the fusion cells, which is determined by allelic differences at *plp-1/sec-9* (30) or *rcd-1* (27–30).

In this study, we identified the *gt69-2* gene from a cross between distantly related N. crassa isolates that segregated for a cell fusion phenotype. The *gt69-2* gene encodes a predicted alpha-1,3-mannosyltransferase that regulates cell wall dissolution during cell fusion and has similarity to the cryptococcal mannosyltransferase 1 (*CMT1*) gene from Cryptococcus neoformans (31). In C. neoformans, Cmt1p catalyzes the transfer of mannose from GDP-mannose to α-1,3-linked mannose disaccharides associated with capsule synthesis. Here, we show that loss-of-function mutations in *gt69-2* resulted in cells that were blocked in cell wall dissolution during cell fusion in N. crassa, a phenotype that was suppressed by loss-of-function mutations in *rfw-1.* Overexpression of *rfw-1* blocked cell fusion and also resulted in a conidial separation phenotype. Population analyses revealed two polymorphic haplotypes at *gt69-2*, with one haplogroup containing a linked *rfw-1* locus, which was absent in members of the second haplotype. These data indicate that the *gt69-2/rfw-1* loci are under balancing selection and provide new mechanisms regulating cell wall remodeling during cell fusion and conidial development in N. crassa.

## RESULTS

### Identification of highly polymorphic loci that segregate with a cell fusion arrest phenotype.

**FIG 1:**
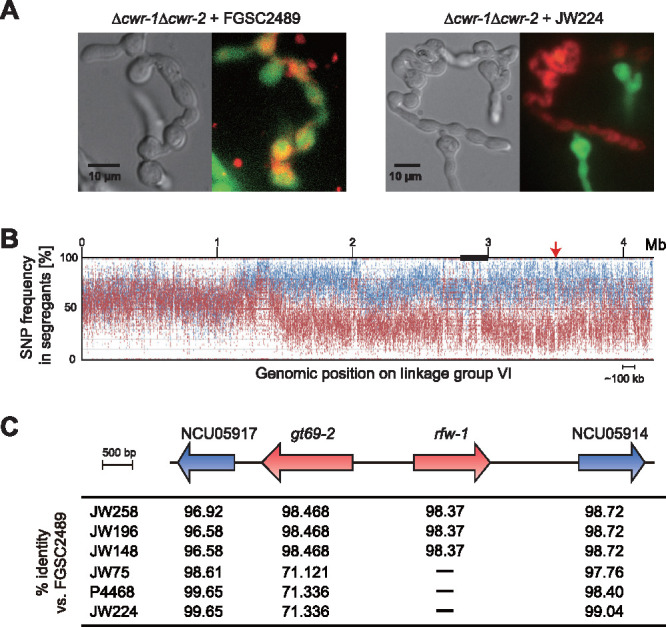
Identification of fusion-associated loci. (A) Examination of cell fusion of GFP-expressing Δ*cwr-1*Δ*cwr-2* germlings (Δ*cwr-1*ΔNCU01381 Δ*cwr-2 his-3*:p*ccg-1-GFP;* Table S1) paired with FM4-64-stained FGSC2489 (the parent of the Δ*cwr-1*Δ*cwr-2* mutant) or Δ*cwr-1*Δ*cwr-2* (GFP) germlings blocked in cell fusion when paired with FM4-64-stained wild isolate JW224 by epifluorescence microscopy. (B) SNP segregation on linkage group VI (from 1.2 Mb to 4.2 Mb) after bulk segregant analysis and sequencing of two pools of genomic DNA from FGSC2489 fusion-compatible versus fusion-incompatible progeny from a cross between FGSC2489 and JW224. Blue line: SNP frequency in pooled segregants compatible with FGSC2489. Red line: SNP frequencies in pooled segregants incompatible with FGSC2489. Black box shows the region of centromere. Red arrow shows the position of *gt69-2* and *rfw-1*. (C) Genomic organization of *gt69-2* (NCU05916) linked loci in FGSC2489 and wild isolates. The percentage identity of the predicted protein sequences from sequenced wild isolates was calculated using FGSC2489 as the reference. The strains lacking NCU05915 (*rfw-1*) are marked with a dash.

Previously, we identified the cell wall remodeling (CWR) loci *cwr-1* and *cwr-2* that regulate cell wall dissolution during somatic cell fusion in N. crassa (29). During somatic cell fusion, hyphae and germlings (germinated asexual spores) that undergo chemotropic interactions, but carry incompatible alleles at *cwr-1* and *cwr-2* loci, fail to degrade the cell wall at the point of contact, thus preventing cytoplasmic mixing (29). Simultaneous deletion of *cwr-1* and *cwr-2* abolishes the block in cell fusion between some strains carrying alternative *cwr* alleles and cells complete the fusion process (29). However, in screening germinated conidia (germlings) from a Δ*cwr-1* Δ*cwr-2* mutant (Δ*cwr-1* ΔNCU01381 Δ*cwr-2*) (Table S1 in the supplemental material) against other wild-type N. crassa isolates, we observed that the Δ*cwr-1* Δ*cwr-2* mutant failed to undergo cell fusion when paired with wild-type strain JW224 (Fig. 1A), suggesting the existence of a second locus that regulated cell wall dissolution during somatic cell fusion. To identify this second locus, we performed bulk segregant analysis (BSA) of progeny from a cross between FGSC2489 (the parental laboratory strain of the Δ*cwr-1* Δ*cwr-2* mutant) and JW224. Progeny segregated into three classes: (i) progeny that underwent chemotropic interactions with FGSC2489 and JW224, but only completed cell fusion with FGSC2489; (ii) progeny that underwent chemotropic interactions with FGSC2489 and JW224, but only completed cell fusion with JW224; and (iii) progeny that failed to fuse with either parent. This third class of progeny was paired with the Δ*cwr-1* Δ*cwr-2* mutant; approximately half of these progeny fused with the Δ*cwr-1* Δ*cwr-2* strain, while the other approximately half did not. Genomic DNA from these two progeny pools of the third class, one pool of progeny that fused with the Δ*cwr-1* Δ*cwr-2* mutant and the second progeny pool that failed to fuse with the Δ*cwr-1* Δ*cwr-2* mutant, was isolated and subjected to whole-genome resequencing. From a comparison of single nucleotide polymorphisms (SNPs) between these two pools, a region spanning approximately 3 Mb on chromosome VI was identified that showed SNP segregation between the Δ*cwr-1* Δ*cwr-2* fusion-compatible and the Δ*cwr-1* Δ*cwr-2* fusion-incompatible pools of progeny (Fig. 1B). Upon further inspection of this 3 Mb region, mapped reads coverage to NCU05915 were significantly lower in Δ*cwr-1* Δ*cwr-2* fusion-incompatible progeny pools compared to Δ*cwr-1* Δ*cwr-2* fusion-compatible progeny pools (Fig. S1A).

10.1128/mBio.00307-21.1 FIG S1 Divergence of NCU05915 and NCU05916 in populations of N. crassa. (A) Mapped sequences were plotted to the reference genome (FGSC2489) region containing the candidate locus associated with cell fusion. The upper panel shows the mapped reads from FGSC2489-compatible progeny pool; the bottom panel shows the mapped reads from FGSC2489-incompatible progeny pool. (B) Genomic organization of *gt69-2*-linked loci in FGSC2489 and wild isolates. The percentage of identity of the predicted protein sequences from sequenced wild isolates was calculated using FGSC2489 as the reference. The strains without NCU05915 (*rfw-1*) are marked with a dash under the gene of NCU05915. Download FIG S1, EPS file, 1.7 MB. Copyright © 2021 Li et al. 2021 Li et al. https://creativecommons.org/licenses/by/4.0/ This content is distributed under the terms of the Creative Commons Attribution 4.0 International license.

10.1128/mBio.00307-21.8 Table S1 Strains of Neurospora crassa used in this study. Download Table S1, DOCX file, 0.02 MB. Copyright © 2021 Li et al. 2021 Li et al. https://creativecommons.org/licenses/by/4.0/ This content is distributed under the terms of the Creative Commons Attribution 4.0 International license.

Using assembled genome sequences of 23 N. crassa isolates (26), we analyzed polymorphisms at NCU05915 and linked loci (NCU05914, NCU05916, and NCU05917) (Fig. S2). Among the 23 strains, alleles at NCU05914 and NCU05917 were highly conserved (>90 amino acid identity) (Fig. 1C, Fig. S1B and S2). In contrast, alleles of NCU05916 showed high sequence diversity and alleles fell into two haplogroups among the 23 wild isolates (Fig. 1C, Fig. S1B and S2). We defined the alleles of NCU05916 with high conservation to FGSC2489 (the laboratory strain; amino acid identity > 96%) as haplogroup I, and alleles that were highly similar to each other but different from haplogroup I alleles, and which included JW224, as haplogroup II (Fig. 1C, Fig. S1 and S2). Interestingly, all the strains within haplogroup II lacked the linked locus NCU05915, while within haplogroup I strains, NCU05915 alleles were highly conserved with above 98% amino acid identity (Fig. 1C, Fig. S1 and S2).

10.1128/mBio.00307-21.2 FIG S2 Phylogenetic trees of NCU05914, NCU05915, NCU05916, and NCU05917. Coding sequences of the indicated genes from 23 N. crassa isolates from a population of Louisiana strains were used to build maximum-likelihood phylogenetic trees. Branch length values are shown below branches (values lower than 0.02 are hidden). Results from 100 bootstrap replicates are given for each node. Black bars indicate substitution rates. Download FIG S2, EPS file, 0.2 MB. Copyright © 2021 Li et al. 2021 Li et al. https://creativecommons.org/licenses/by/4.0/ This content is distributed under the terms of the Creative Commons Attribution 4.0 International license.

**FIG 2:**
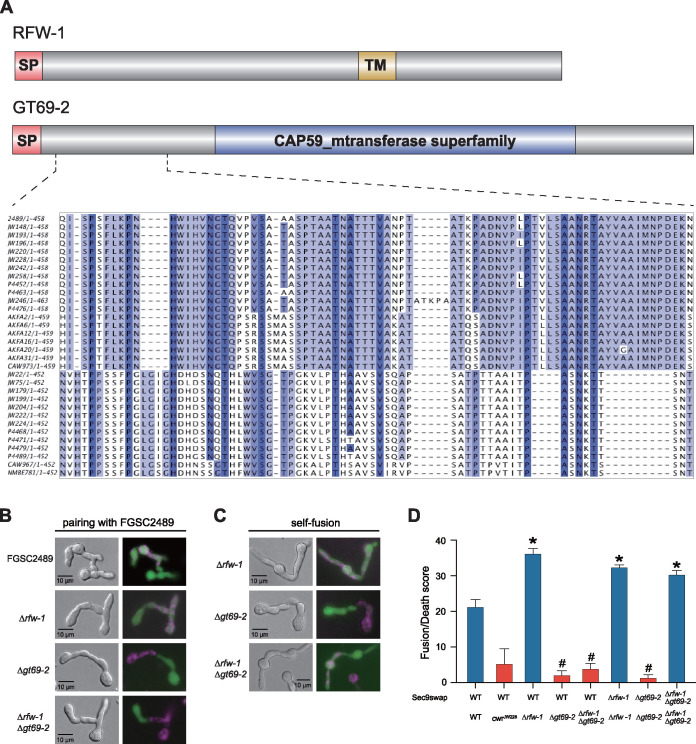
Cell fusion is blocked in the Δ*gt69-2* cells, but is restored in Δ*rfw-1*Δ*gt69-2* germlings. (A) Schematic drawing of the NCU05915 (RFW-1) and NCU05916 (GT69-2) proteins and amino acid alignment of the highly divergent region with *gt69-2* orthologs from N. crassa and N. discreta wild isolates. SP, signal peptide; TM, transmembrane domain; CAP59_mtransferase superfamily, alpha-1,3-mannosyltransferase catalytic domain. (B) Assays of cell fusion with FGSC2489 by epifluorescence microscopy. FM4-64-stained FGSC2489, Δ*rfw-1*, Δ*gt69-2*, or Δ*rfw-1*Δ*gt69-2* germlings paired with FGSC2489 expressing cytoplasmic GFP. (C) Assay of self-fusion phenotype in the indicated strains by epifluorescence microscopy. Self pairings of Δ*rfw-1*, Δ*gt69-2*, or Δ*rfw-1*Δ*gt69-2* where half of the germling were stained with FM4-64 and the other half expressed cytoplasmic GFP. (D) Quantification of cell fusion via flow cytometry using a cell death assay activated upon cell fusion (29); sec9swap indicates that strains contain an incompatible allele of *sec-9*. FGSC2489 + FGSC2489 (WT + WT) pairing is the positive control for cell fusion and shows a high cell death score. FGSC2489 (WT) + *cwr*^JW228^ is a negative control (blocked in cell fusion) showing a low cell death score (29). *, *P* value < 0.0001 versus negative control (WT + *cwr*^JW228^); #, *P* value < 0.001 versus positive control (WT + WT).

NCU05916 encodes a predicted 457-amino acid (aa) alpha-1,3-mannosyltransferase with a conserved “CAP59_mtransfer” protein domain (Fig. 2A), which showed 36% identity to Cryptococcus neoformans Cmt1p (Cryptococcus mannosyltransferase 1), an enzyme with alpha-1,3-mannosyltransferase activity (31). NCU05916 has been designated *gt69-2* to reflect its predicted biochemical activity as a glycosyl transferase member in family 69 (http://www.cazy.org/GT69.html). NCU05915 encodes a predicted 367-amino acid protein lacking identifiable functional domains except a transmembrane domain (Fig. 2A); we named NCU05915 as *regulator of cell fusion and cell wall remodeling1* (*rfw-1*) for its phenotype (see below). Both NCU05915 and NCU05916 contained a predicted N-terminal signal peptide (SP) (Fig. 2A). Alignment of GT69-2 from 23 N. crassa isolates showed a region in the N terminus that was highly divergent (HD) between the two different haplogroups (Fig. 2A).

### Cell fusion deficient phenotype of Δ*gt69-2* is suppressed by mutations in *rfw-1*.

To determine whether *gt69-2* and/or *rfw-1* was responsible for cell fusion arrest, we generated Δ*gt69-2* and Δ*rfw-1* single deletion mutants, and a Δ*rfw-1*Δ*gt69-2* double deletion mutant by replacing *gt69-2*, *rfw-1*, or the whole region containing both *rfw-1* and *gt69-2* with a hygromycin B-resistance cassette in an FGSC2489 background (see the Materials and Methods) (Fig. S3A and B). Cell fusion assays were performed by pairing FM4-64-stained mutant germlings with FGSC2489 germlings expressing cytoplasmic green fluorescent protein (GFP). The Δ*gt69-2* and Δ*rfw-1*Δ*gt69-2* germlings underwent chemotropic interactions, but failed to complete cell fusion and cytoplasmic mixing with FGSC2489 germlings (Fig. 2B). In contrast, the Δ*rfw-1* mutant showed a wild-type cell fusion phenotype when paired with FGSC2489. These data indicated that *gt69-2* was required for successful cell fusion with its wild-type parental strain.

To assess self-fusion defects, we crossed cytoplasmic GFP into the Δ*gt69-2*, Δ*rfw-1*, and Δ*rfw-1*Δ*gt69-2* mutants. Similar to results obtained in pairings with the parental strain (FGSC2489), self fusion was observed in Δ*rfw-1* cells but was blocked in Δ*gt69-2* cells (Fig. 2C). However, to our surprise, the Δ*rfw-1*Δ*gt69-2* double mutant cells underwent self fusion (Fig. 2C). These data indicated that the cell fusion arrest observed when the Δ*rfw-1*Δ*gt69-2* double mutant was paired with its isogenic parent FGSC2489 (with functional alleles of *gt69-2* and *rfw-1*) was alleviated in Δ*rfw-1*Δ*gt69-2* self pairings.

To confirm that a deletion of *rfw-1* suppresses the cell fusion defect of Δ*gt69-2*, we generated a second double mutant by introducing a Δ*rfw-1* deletion into a Δ*gt69-2* mutant by replacing *rfw-1* with a nourseothricin-resistance cassette (see the Materials and Methods) (Fig. S3A and B). This independently derived double mutant (ΔNCU05915 Δ*gt69-2*) (Table S1) showed an identical slant phenotype to the Δ*rfw-1* Δ*gt69-2* mutant (Fig. S3C) and, identical to the Δ*rfw-1*Δ*gt69-2* mutant, underwent fusion in self pairings but not when paired with FGSC2489 (Fig. S3D). These data supported the original observation that deletion of *rfw-1* suppressed the cell fusion defects of the Δ*gt69-2* mutant.

10.1128/mBio.00307-21.3 FIG S3 Verification of mutants, growth, and cell fusion phenotypes. (A) Schematic drawing of the target genes for deletion, resistance marker (HYG or NAT) cassette, and the positions/directions of the PCR primers. (B) PCR verification of various mutants. The labeled primer pairs (Table S2) were used for PCR analysis with genomic DNA of the wild type FGSC2489 (lane 1) and genomic DNA from the indicated mutants (lanes 2 to 13). Top left: about a 1-kbp PCR product of the resistance marker (HYG or NAT) is observed in the mutants, but not in the FGSC2489 parental strain. Top right: primer pairs (Table S2) for the targeted deletions were used to confirm the genotype of the mutants. About a 1-kbp PCR product was observed in FGSC2489, but the target genes were not amplified in the mutants. Bottom left: for each mutant, a forward primer beyond the 5′ flank and a reverse primer in the resistance marker (Table S2) were used to confirm that the target gene/region was replaced by the resistance marker. About a 1.5-kbp PCR fragment was observed in the mutants, but not in FGSC2489. Bottom right: a forward primer for the resistance marker and a reverse primer beyond the 3′ flank (Table S2) were used to confirm that the target gene/region was replaced by the resistance marker. About a 1.5-kbp PCR fragment was observed in mutants, but not in FGSC2489. (C) The indicated strains were grown in slant tubes for 7 days. The Δ*gt69-2* mutant showed shorter aerial hyphae compared to FGSC2489, but both the ΔNCU05915 Δ*gt69-2* and Δ*rfw-1*Δ*gt69-2* mutants had a similar phenotype to FGSC2489, indicating that the short aerial hyphae phenotype of Δ*gt69-2* was suppressed by deletion of *rfw-1*. (D) Examination of cell fusion of FM4-64-stained ΔNCU05915 Δ*gt69-2* germlings paired with GFP-expressing ΔNCU05915 Δ*gt69-2* or FGSC2489 germlings by epifluorescence microscopy. (E) Strain showing complementation of the Δ*gt69-2* mutant phenotype by introduction of a GFP-tagged *gt69-2* construct driven by the *ccg-1* promoter. Strains showing that the introduction of GFP-tagged *gt69-2^JW224^* allele complemented the Δ*gt69-2* growth phenotype in the FGSC2489 background. The indicated strains were grown in slant tubes for 7 days. Download FIG S3, EPS file, 1.1 MB. Copyright © 2021 Li et al. 2021 Li et al. https://creativecommons.org/licenses/by/4.0/ This content is distributed under the terms of the Creative Commons Attribution 4.0 International license.

To quantify cell fusion frequencies in the mutants relative to wild-type cells, we utilized a flow cytometry method based on a robust postfusion death response in germinated spores that is mediated by genetic differences at *sec-9* (29, 30). In brief, FGSC2489 and mutant strains were engineered to carry *sec-9^GRD2^* at the native *sec-9* locus. When germlings carrying incompatible *sec-9* alleles undergo cell fusion, cell death is induced within 20 min, which can be used as a proxy for cell fusion frequency using vital dyes and flow cytometry (29, 30). FGSC2489 + FGSC2489^sec-9swap^ pairings were used as a positive control and showed a high death rate (∼22%), while a negative-control pairing between cells unable to complete cell fusion (FGSC2489 with *cwr-1*^JW228^ + FGSC2489^sec-9swap^) showed a low death frequency (∼5%) (Fig. 2D), a value consistent with that previously reported (29). As predicted by microscopic analyses, the Δ*gt69-2 *+ FGSC2489^sec-9swap^ pairings, the Δ*gt69-2* + Δ*gt69-2*^sec-9swap^ pairings, and the Δ*rfw-1*Δ*gt69-2 *+ FGSC2489^sec-9swap^ pairings all showed a low death frequency (2 to 5%) (Fig. 2D), consistent with a block in cell fusion. In line with the microscopy results, the Δ*rfw-1 *+ FGSC2489^sec-9swap^ pairings and the Δ*rfw-1* + Δ*rfw-1*^sec-9swap^ pairings both showed a high level of death frequency, showing that cells lacking *rfw-1* are not affected in cell fusion (Fig. 2D). The Δ*rfw-1*Δ*gt69-2* + Δ*rfw-1*Δ*gt69-2*^sec-9swap^ self-pairings also showed a high death frequency (Fig. 2D), confirming that the lack of *rfw-1* suppressed the cell fusion defect of the Δ*gt69-2* mutant. Additionally, these data also showed that neither GT69-2 nor RFW-1 was essential for cell fusion, as Δ*rfw-1*Δ*gt69-2* germlings showed self-fusion frequencies that were slightly higher than parental WT germlings (Fig. 2D).

### Genetic interactions between *gt69-2* and *rfw-1*.

**FIG 3:**
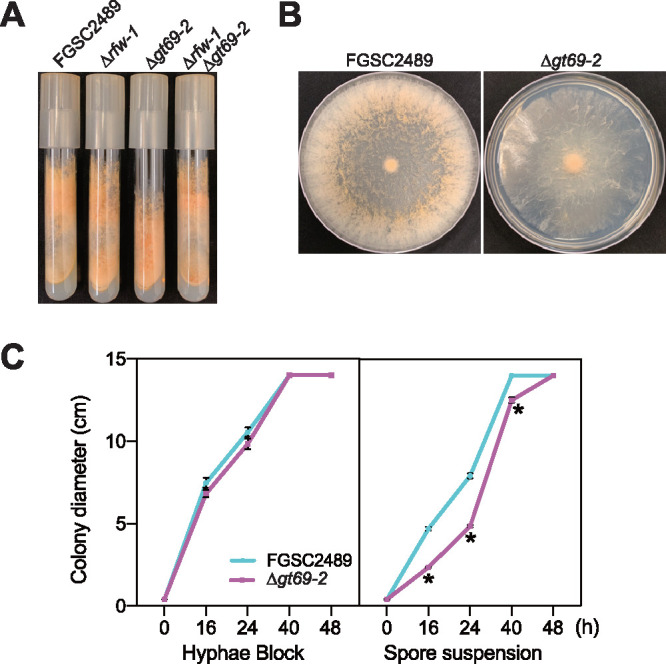
Phenotypic assays of Δ*gt69-2*. (A) The indicated strains were grown in slant tubes for 7 days. Δ*rfw-1* and Δ*rfw-1*Δ*gt69-2* have a similar growth phenotype to FGSC2489, but the Δ*gt69-2* mutant shows a shorter height of aerial hyphae compared to FGSC2489. (B) Spores from FGSC2489 and Δ*gt69-2* were inoculated onto the center of a petri dish and photographs of the colonies were taken after 48 h of growth. The Δ*gt69-2* mutant showed slower growth and fewer aerial hyphae compared to FGSC2489. (C) The colony diameter of FGSC2489 and Δ*gt69-2* strains was measured after 48 h of growth when inoculated from a hyphal plug versus a conidial spore suspension. *n* = 4; *, *P* value < 0.0001 versus FGSC2489.

The Δ*gt69-2* mutant showed a lower height of aerial hyphae compared to FGSC2489 (Fig. 3A), a phenotype that has been observed in other cell fusion mutants (21, 32, 33). However, this phenotype was not observed in the Δ*rfw-1* or Δ*rfw-1*Δ*gt69-2* mutant strains, indicating that, analogously to the cell fusion process, the short aerial hyphae phenotype of Δ*gt69-2* was suppressed by deletion of *rfw-1*. To test whether the Δ*gt69-2* mutant showed a lower growth rate, we inoculated hyphal plugs or conidial suspensions of each strain on Vogel’s minimal medium (VMM) agar plates and measured the diameters of colonies up to 2 days postinoculation. When a conidial suspension was inoculated onto plates, the Δ*gt69-2* mutant showed a smaller colony diameter and fewer aerial hyphae compared to FGSC2489 (Fig. 3B and C). By plotting colony diameter over time, the Δ*gt69-2* showed a lower growth rate for 24 h, consistent with a lag in colony establishment, a phenotype that has also been observed in other cell fusion mutants (21) (Fig. 3C). In contrast, with hyphal plug inoculations—that is, after the colony was already established—the Δ*gt69-2* mutant and FGSC2489 showed a similar growth rate (Fig. 3C). These data indicated that *gt69-2* was dispensable for growth rate of a mycelial colony, but important for colony establishment via germling fusion.

**FIG 4:**
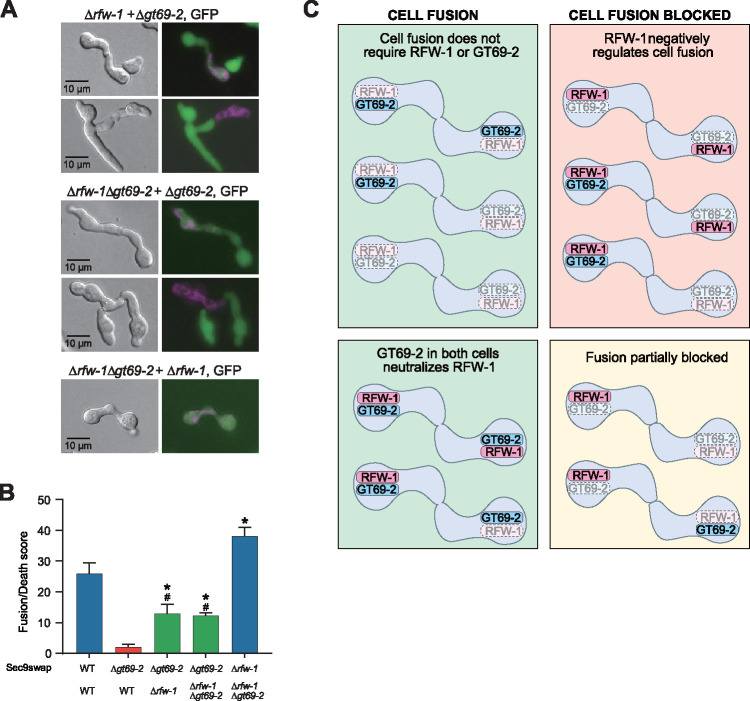
Cell fusion frequencies between (Δ*rfw-1* + Δ*gt69-2*), (Δ*rfw-1* + Δ*rfw-1*Δ*gt69-2*), or (Δ*gt69-2* + Δ*rfw-1*Δ*gt69-2*) germling pairs. (A) Assay of cell fusion of indicated germling pairs by epifluorescence microscopy; (Δ*rfw-1* + Δ*gt69-2*) and (Δ*gt69-2* + Δ*rfw-1*Δ*gt69-2*) germling pairs showed a mixture of cell fusion phenotypes. (B) Quantification of cell fusion via flow cytometry. sec9swap indicates that the strains contain an incompatible allele of *sec-9*. WT (FGSC2489) + WT (FGSC2489) pairing is the positive control for cell fusion, while WT (FGSC2489) + Δ*gt69-2* is the negative control for cell fusion (red column). Both (Δ*rfw-1* + Δ*gt69-2*) and (Δ*gt69-2* + Δ*rfw-1*Δ*gt69-2*) germling pairs showed an intermediate value of cell death scores (green columns). *, *P* value < 0.001 versus negative control; #, *P* value < 0.001 versus positive control; *n* = 3. (C) Schematic showing the cell fusion phenotype of various germling pair combinations. Top left panel: when paired cells lack *rfw-1* or both *rfw-1* and *gt69-2*, cell fusion is not affected. Bottom left panel: when paired cells have *gt69-2*, with or without *rfw-1*, successful cell fusion occurs. Top right panel: for pairs of cells that have *rfw-1*, but lack *gt69-2* in one partner cell, fusion is completely blocked. Bottom right panel: pairing of cells with functional *rfw-1* but lacking *gt69-2* shows a partially blocked cell fusion phenotype.

The cell fusion defect of the Δ*gt69-2* mutant was suppressed in the Δ*rfw-1*Δ*gt69-2* double mutant (Fig. 2C). To further explore the genetic interactions between *rfw-1* and *gt69-2*, we assayed the cell fusion phenotype of strains carrying different combinations of *rfw-1* and *gt69-2* deletions (wild-type alleles present in the respective strains are shown with a superscript plus [+] sign) by microscopy and by flow cytometry (Fig. 4A and B). As shown in Fig. 2C, Δ*rfw-1*Δ*gt69-2* + Δ*rfw-1*Δ*gt69-2* germlings undergo cell fusion, as did pairings between Δ*rfw-1 gt69-2*^+^+ Δ*rfw-1*Δ*gt69-2* germlings (Fig. 4A), which was confirmed using flow cytometry (Fig. 4B). However, Δ*rfw-1 gt69-2*^+^ + *rfw-1*^+^Δ*gt69-2* pairings showed a mixed cell fusion phenotype (Fig. 4A and B), where some pairs underwent cell fusion while others were blocked. Similarly, pairings between *rfw-1*^+^Δ*gt69-2* + Δ*rfw-1*Δ*gt69-2* pairs also showed a mixed cell fusion phenotype and reduced fusion frequency (Fig. 4A and B). These data indicated that in cells that lacked *gt69-2* but contained *rfw-1*, cell fusion was fully or partially blocked. For successful fusion, *gt69-2* was required in both cells if *rfw-1* was present in either one or both cells. A summary of the cell fusion phenotypes of different combinations of *rfw-1* and *gt69-2* mutants is shown in Fig. 4C.

### Cells lacking *gt69-2* affect oscillation of MAK-2 and are blocked in cell wall dissolution.

**FIG 5:**
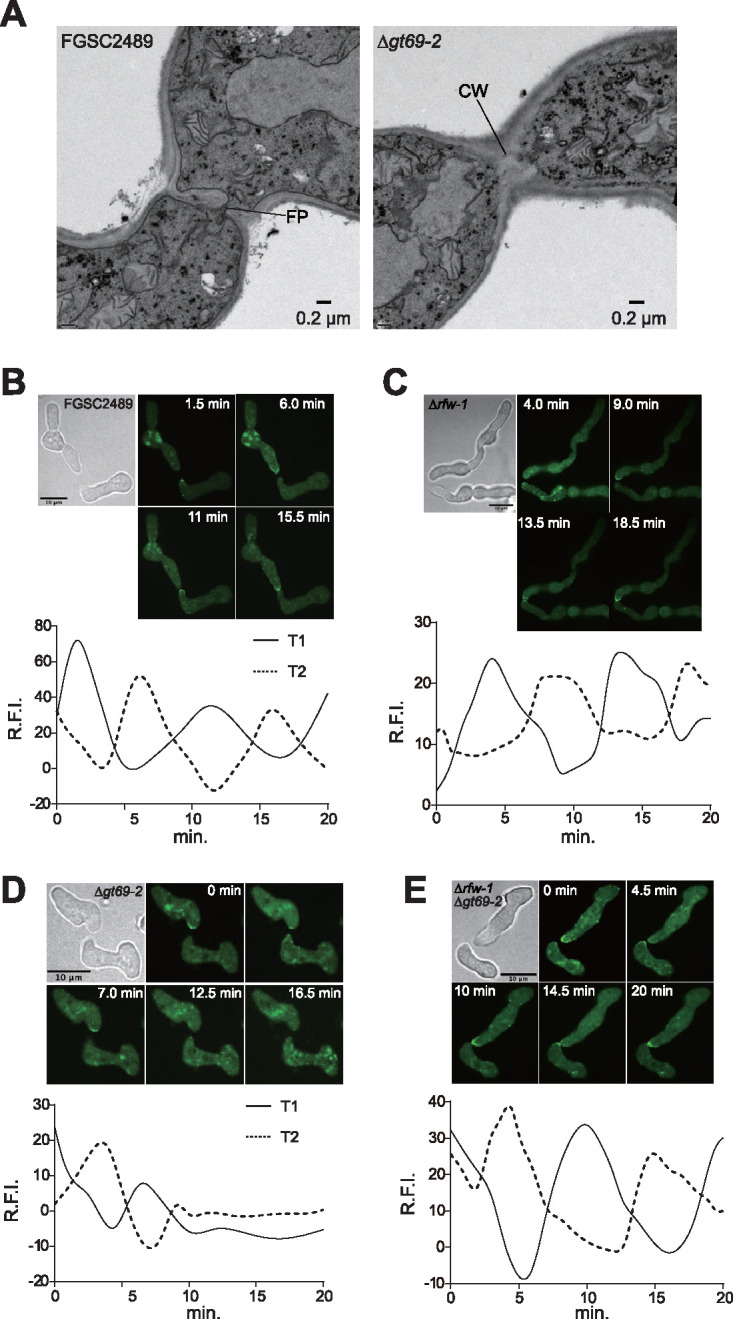
Fusion phenotype of Δ*gt69-2* germlings shows a block in cell wall dissolution. (A) Transmission electron microscopy of FGSC2489 or Δ*gt69-2* germlings undergoing self-fusion. FP, fusion pore; CW, cell wall. (B) Microscopic and graphic representation of MAK-2-GFP localization in FGSC2489 in germling pairs undergoing chemotropic interactions. (C) Microscopic and graphic representation of MAK-2-GFP localization in Δ*rfw-1* germling pairs undergoing chemotropic interactions. (D) Microscopic and graphic representation of MAK-2-GFP localization in Δ*gt69-2* germling pairs undergoing chemotropic interactions. (E) Microscopic and graphic representation of MAK-2-GFP localization in Δ*rfw-1*Δ*gt69-2* germling pairs undergoing chemotropic interactions. T1 = CAT tip of germling one; T2 = CAT tip of germling two. The *y* axis shows the ratio of relative fluorescence intensity (R.F.I.) in the interaction zone compared to background. The *x* axis shows time (min). Panels show representative experiments; *n* = 4.

To assess when the cell fusion defect occurred in Δ*gt69-2* cells, we first used transmission electron microscopy to determine whether the fusion defect in Δ*gt69-2* cells was due to a failure in cell wall dissolution or in membrane merger. In FGSC2489 + FGSC2489 samples, cell wall and plasma membrane dissolution at the point of contact between germling fusion pairs was easily observed (Fig. 5A). In contrast, in Δ*gt69-2* + Δ*gt69-2* pairings, we failed to find cell wall dissolution at contact points (Fig. 5A), and accumulation of cell wall material at cell-cell contact sites was not observed, in contrast to cell pairings between incompatible *cwr* strains (29). These data indicated that the block of cell fusion in Δ*gt69-2* mutant was caused by failure of cell wall breakdown upon contact between Δ*gt69-2* cells.

During chemotropic interactions between compatible cells, the mitogen-activated protein kinase (MAPK) signal transduction protein complex (NRC-1, MEK-2, MAK-2, and the scaffold protein HAM-5) are recruited to conidial anastomosis tubes (CATs) (19). The MAK-2 complex assembles and disassembles at CAT tips every 8 to 10 min; chemical inhibition of the phosphorylation activity of MAK-2 results in immediate cessation of chemotropic growth (20). A second protein complex bearing SOFT (SO) also assembles and disassembles at CAT tips, but perfectly out of phase with the MAK-2 complex (20). FGSC2489 (MAK-2-GFP) + FGSC2489 (SOFT-dsRED) cells display oscillation of MAK-2 and SOFT to CATs during chemotropic interactions until physical contact. Previously, we showed that in cell pairings between incompatible *cwr* strains, MAK-2 and SO continued to oscillate at the contact point, consistent with an inability of *cwr* incompatible cells to transit from chemotropic growth to cell wall dissolution (29).

To further explore the block in self cell fusion in the Δ*gt69-2* cells, we analyzed MAK-2-GFP localization in Δ*rfw-1*(*mak-2-gfp*) germlings, in Δ*gt69-2* (*mak-2-gfp*) germlings, and in Δ*rfw-1*Δ*gt69-2*(*mak-2-gfp*) germlings. In wild-type pairings, MAK-2-GFP shows dynamic localization to CATs during chemotropic interactions, localizing to one CAT tip while disappearing from its partner cell every ∼4.5 min (Fig. 5B). Consistent with microscopic observations showing wild-type levels of cell fusion, the Δ*rfw-1* cells showed normal dynamics of MAK-2 oscillation during chemotropic interactions (Fig. 5C). In pairings between Δ*gt69-2* cells, oscillation of MAK-2 was observed during chemotropic interactions, but when Δ*gt69-2* germlings were in close proximity, MAK-2 localization to CATs was no longer observed (Fig. 5D). Additionally, MAK-2 localization at the contact point between Δ*gt69-2* germlings was not observed, which is apparent in wild-type pairings. These data indicated that Δ*gt69-2* germlings were affected during interactions when cells were in close proximity and in subsequent cell wall dissolution. Importantly, normal MAK-2-GFP dynamics during chemotropic interactions were restored in self pairings of Δ*rfw-1*Δ*gt69-2* germlings, consistent with the suppression of the cell fusion defect of the Δ*gt69-2* cells by deletion of *rfw-1* (Fig. 5E).

### GT69-2 and RFW-1 localization, overexpression phenotypes, and sensitivity to cell wall stress.

**FIG 6:**
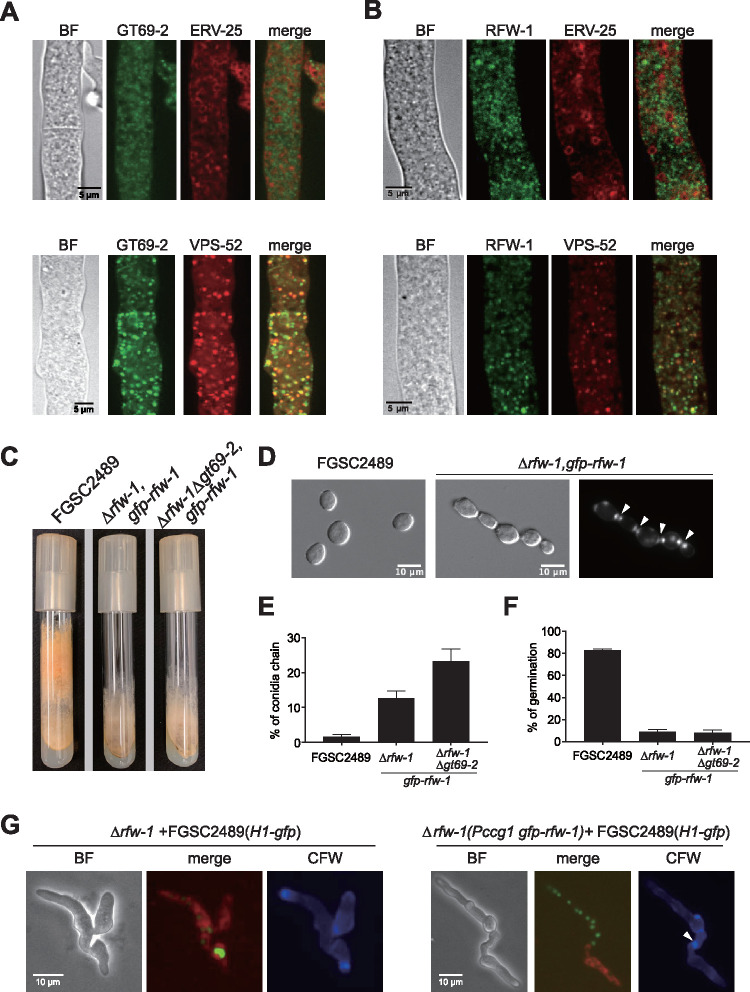
Cellular localization of GT69-2 and RFW-1 and phenotype of strains overexpressing *rfw-1*. (A) Upper panel shows confocal images of heterokaryons coexpressing GFP-GT69-2 and the ER marker mCherry-ERV-25; bottom panel shows confocal images of heterokaryons coexpressing GFP-GT69-2 and the Golgi marker mCherry-VPS-52 imaged by confocal microscopy. (B) Cellular localization of RFW-1. Upper panel shows confocal images of heterokaryons coexpressing GFP-RFW-1 and ER marker mCherry-ERV-25; bottom panel shows confocal images of heterokaryons coexpressing GFP-RFW-1 and the Golgi marker mCherry-VPS-52. (C) Slant tube phenotype of the indicated strains grown for 7 days. (D) Strains overexpressing *rfw-1* (p*ccg-1gfp-rfw-1*) showing a conidial separation defect. Left panel shows free conidia from FGSC2489. Middle panel shows the conidial separation defect observed in the Δ*rfw-1* (p*ccg-1gfp-rfw-1*) strain. Right panel shows conidial septa stained by calcofluor white. Arrows show the double-doublet staining of septa between conidia. (E) Frequency of conidial chains in cultures of the indicated strains; for example, 13% conidial chains means that 13 conidial chains were observed in a population of 100 conidia. *n* = 4. (F) Percentage of germination of conidia at 3 h after conidial suspensions from FGSC2489 and Δ*rfw-1* (p*ccg-1gfp-rfw-1*) were inoculated on VMM plates. (G) Microscopic analyses of cell fusion of Δ*rfw-1* or Δ*rfw-1* (p*ccg-1gfp-rfw-1*) paired with an FGSC2489 strain expressing histone 1-GFP (H1-GFP). Δ*rfw-1* and Δ*rfw-1* (p*ccg-1gfp-rfw-1*) germlings were stained with FM4-64. Cytoplasmic mixing was observed in Δ*rfw-1 *+ FGSC2489 (H1-*gfp*) pairings (left panel) but not in Δ*rfw-1* (p*ccg-1gfp-rfw-1*) + FGSC2489 (H1-*gfp*) pairings (right panel). Calcofluor white staining showed undissolved cell wall (arrowheads) at the contact point between Δ*rfw-1* (p*ccg-1gfp-rfw-1*) and FGSC2489 (H1-*gfp*) cells.

Both GT69-2 and RFW-1 have predicted signal peptides. To characterize the subcellular localization of GT69-2 and RFW-1, we fused GFP to the N-terminal region of the predicted proteins immediately after the predicted signal peptides. The GFP-fused *gt69-2* and *rfw-1* were driven by the *ccg-1* promoter and expressed in Δ*gt69-2* and Δ*rfw-1* cells, respectively; GFP fluorescence was not observed in constructs using the *gt69-2* or *rfw-1* native promoters. The *ccg-1*-regulated *gfp-gt69-2* construct fully complemented the growth and cell fusion defects of the Δ*gt69-2* mutant (Fig. S3E). Both GFP-GT69-2 and GFP-RFW-1 showed a similar subcellular localization pattern as numerous fluorescent punctate structures in hyphal compartments (Fig. 6A and B), with a similar localization pattern in germlings (Fig. S4). It is likely that increased protein levels from *ccg-1*-driven *gt69-2* and *rfw-1* expression resulted in a more abundant localization to Golgi. Localization of GFP-GT69-2 or GFP-RFW-1 to puncta within the cell did not change in germlings undergoing chemotropic interactions or cell fusion. To determine which organelles the puncta were, we coexpressed GFP-GT69-2 or GFP-RFW-1 with the Golgi marker mCherry-VPS-52 or the ER marker mCherry-ERV-25 in heterokaryotic strains. Colocalization of GFP-GT69-2 or GFP-RFW-1 with the ER marker ERV-25 was not observed, however, many of the GFP-GT69-2 and GFP-RFW-1 puncta colocalized with mCherry-VPS-52 (Fig. 6A and B). These data suggested that the punctate structures to which GFP-GT69-2 and GFP-RFW-1 localized were Golgi compartments.

10.1128/mBio.00307-21.4 FIG S4 Slant tube phenotype and RFW-1-GFP localization in Δ*rfw-1* (p*rfw-1gfp-rfw-1*) and Δ*rfw-1* (p*ccg-1gfp-rfw-1*) strains. (A) Slant tube phenotype of the indicated strains was evaluated after 7 days of growth. (B) GFP signal of the indicated strains was examined using epifluorescence microscopy. Upper panel: when driven by the native promoter, GFP signals of *gfp*-*rfw-1* were not observed in conidia and germlings. Lower panel: localization of RFW-1-GFP in Δ*rfw-1* (p*ccg-1gfp-rfw-1*) germlings. Download FIG S4, EPS file, 0.4 MB. Copyright © 2021 Li et al. 2021 Li et al. https://creativecommons.org/licenses/by/4.0/ This content is distributed under the terms of the Creative Commons Attribution 4.0 International license.

The Δ*rfw-1* mutant did not show obvious growth or cell fusion defects. However, when GFP-RFW-1 driven by the *ccg-1* promoter was expressed in Δ*rfw-1* or Δ*rfw-1*Δ*gt69-2* cells, the resulting strains Δ*rfw-1* (p*ccg-1gfp-rfw-1*) and Δ*rfw-1*Δ*gt69-2* (p*ccg-1gfp-rfw-1*) showed significantly less and shorter aerial hyphae and numerous conidial chains with unreleased conidia (Fig. 6C to E). Calcofluor white staining showed the unreleased conidia were separated by two complete septa (Fig. 6D), suggesting that the conidial chains were caused by failure of the digestion of the connective material between these two septa. The Δ*rfw-1* (p*ccg-1gfp-rfw-1*) and Δ*rfw-1*Δ*gt69-2* (p*ccg-1gfp-rfw-1*) strains were also delayed in conidial germination. Three hours after plating a conidial suspension onto VMM agar plates, the majority of FGSC2489 conidia germinated, while the majority of Δ*rfw-1* (p*ccg-1gfp-rfw-1*) and Δ*rfw-1*Δ*gt69-2* (p*ccg-1gfp-rfw-1*) conidia remained ungerminated (Fig. 6F). When GFP-RFW-1 was driven by its native promoter in Δ*rfw-1* cells, a GFP signal was not detected, nor were conidial separation and germination defects observed in the Δ*rfw-1* (p*rfw-1gfp-rfw-1*) strain, in contrast to the Δ*rfw-1* (p*ccg-1gfp-rfw-1*) and Δ*rfw-1*Δ*gt69-2* (p*ccg-1gfp-rfw-1*) strains (Fig. S4).

To test whether overexpression of *rfw-1* also resulted in cell fusion defects, we paired FM4-64-stained Δ*rfw-1* (p*ccg-1gfp-rfw-1*) cells with FGSC2489 expressing histone 1-GFP. As shown in Fig. 6G, cytoplasmic mixing was not observed between Δ*rfw-1* (p*ccg-1gfp-rfw-1*) cells + FGSC2489 expressing histone 1-GFP (Fig. 6G). The cell wall, as shown by staining with calcofluor white, was also observed at the contact points. In contrast, cytoplasmic mixing and cell wall breakdown occurred in pairings between the Δ*rfw-1* mutant and FGSC2489 (H1-GFP) (Fig. 6G). These data indicated that, in addition to a conidial separation defect, cell fusion between Δ*rfw-1* (p*ccg-1gfp-rfw-1*) and FGSC2489 was blocked.

The *gt69-2* locus encodes an alpha-1,3-mannosyltransferase predicted to transfer a mannosyl group to either a carbohydrate or a lipid. We therefore hypothesized that loss of *gt69-2* might affect aspects of the cell wall biosynthesis. To test this hypothesis, we assessed growth of Δ*rfw-1*, Δ*gt69-2*, and Δ*rfw-1*Δ*gt69-2* mutants on agar media containing different cell wall stress drugs, including the β-1,3-glucan synthase inhibitor caspofungin and two different anionic dyes that bind chitin and block chitin-glucan cross-linking, calcofluor white and Congo red. Similar to the parental strain FGSC2489, the Δ*rfw-1* and Δ*rfw-1*Δ*gt69-2* mutants were mildly sensitive to all three drugs (Fig. S5). Consistent with conidial inoculations, the Δ*gt69-2* mutant showed a slight growth defect in drug-free medium. However, these defects were not exacerbated on caspofungin, calcofluor white, or Congo red, indicating that the absence of *gt69-2* did not result in major cell wall defects.

10.1128/mBio.00307-21.5 FIG S5 Assay for sensitivity to cell wall stresses in FGSC2489 versus various *rfw-1* and *gt69-2* mutant combinations. (A) A 1:5 serial dilution from ∼5,000 spores per spot to ∼8 spores per spot was performed on the indicated strains. All agar media contained VMM and FGS to force colonial growth. Plates were incubated at 30°C for 5 days. Drug concentrations: 1.3 μg/ml caspofungin, 1.5 mg/ml calcofluor white, and 1 mg/ml Congo red. Download FIG S5, EPS file, 0.3 MB. Copyright © 2021 Li et al. 2021 Li et al. https://creativecommons.org/licenses/by/4.0/ This content is distributed under the terms of the Creative Commons Attribution 4.0 International license.

### Alleles at *gt69-2* and *rfw-1* show evidence of balancing selection.

**FIG 7:**
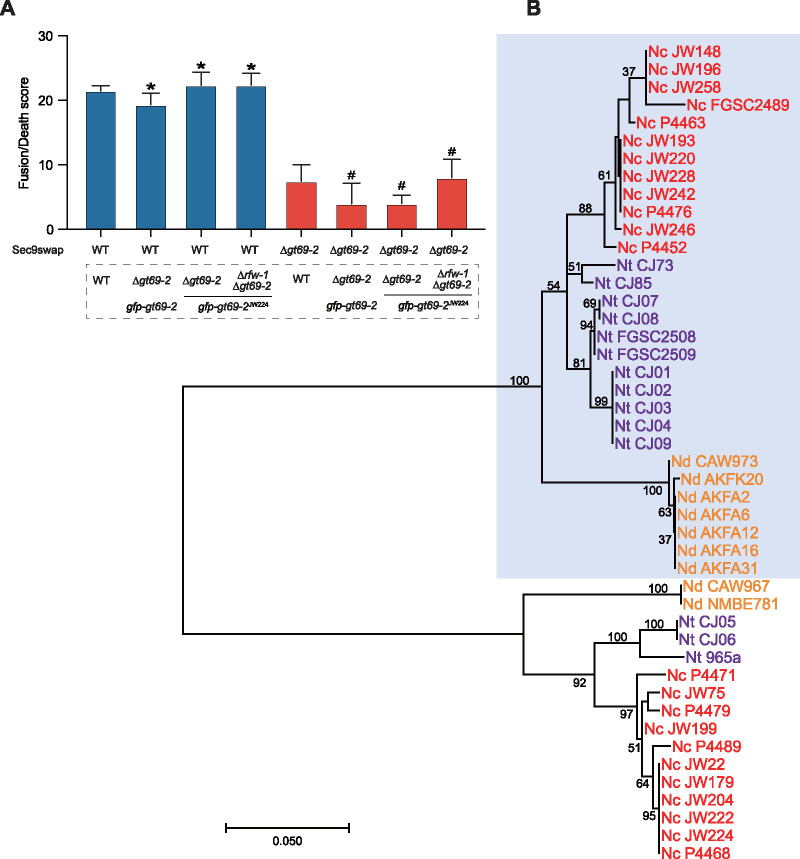
Haplotypes of *gt69-2* alleles in populations of *Neurospora* and cell fusion frequency of germling pairs containing alternate *gt69-2* alleles. (A) Flow cytometry results of sec9swap strains with alleles from the different haplogroups of *gt69-2*. WT (FGSC2489) + WT (FGSC2489) pairings were a positive control for cell fusion and showed a high cell death score; WT (FGSC2489) + Δ*gt69-2* pairings were the negative control and showed a low cell death score due to a block cell fusion. *, *P* value < 0.0001 versus negative control; #, *P* value < 0.001 versus positive control; *n* = 3. (B) Phylogenetic analyses of *gt69-2* orthologs in *Neurospora* species show transspecies polymorphisms. Amino acid sequences of *gt69-2* from indicated isolates were used to build a maximum-likelihood phylogenetic tree. Results from 100 bootstrap replicates are shown beside branches. Strains of the same species are shown in identical colors. Nc, Neurospora crassa; Nd, *Neurospora discreta*; Nt, *Neurospora tetrasperma*. Light blue boxed *gt69-2* alleles have linked *rfw-1* alleles.

Genes that regulate allorecognition, such as the major histocompatibility complex (MHC) in humans, the *S* locus in plants, allorecognition loci in colonial ascidians, and heterokaryon incompatibility loci in fungi, often show evidence of balancing selection, which includes the presence of discrete haplotypes in populations, nearly equal frequency of allelic classes in population samples, and transspecies polymorphisms (26, 34–36). In N. crassa populations, *gt69-2* alleles fell into two discrete haplotypes, suggesting a role in allorecognition (Fig. 2A). In strains containing *rfw-1*, the gene was always linked with *gt69-2* and was highly conserved among isolates. Phylogenetic trees were constructed to test whether allelic polymorphisms at *rfw-1* (NCU05915) and *gt69-2* (NCU05916) were retained among different *Neurospora* species. Consistent with their potential role in allorecognition, the *gt69-2* alleles clustered by haplogroup rather than by species (Fig. 7B). The *gt69-2* alleles from Neurospora discreta and Neurospora tetrasperma isolates grouped into the same two N. crassa haplogroups. Similar to N. crassa, the haplogroup I *gt69-2* alleles in both *N. discreta* and *N. tetrasperma* were linked to *rfw-1*, while species of all strains within haplogroup II lacked *rfw-1*. The transspecies polymorphisms observed in the *gt69-2* alleles suggested that this locus was under balancing selection and that allelic polymorphisms at this locus predates divergence of these species. We tested this hypothesis by calculating the Tajima’s D values for the *gt69-2* alleles. The high, positive, and significant Tajima’s D values calculated for *gt69-2* (Tajima’s D = 2.07708; *P* < 0.05), but not NCU05914 (Tajima’s D = 0.73738; *P* > 0.1) or NCU05917 (Tajima’s D = 1.07540; *P* > 0.1), indicated that *gt69-2* is under balancing selection in *Neurospora* species.

To assess whether allelic polymorphisms were present in other species of fungi, we analyzed the *gt69-2* and *rfw-1* homologs among various species of *Fusarium*, in particular, Fusarium oxysporum, as genome sequences for multiple isolates are available (Table S3). In Fusarium species, most strains have more than one paralog of *gt69-2* and *rfw-1* (Fig. S6). However, in strains of different species of *Fusarium*, if *rfw-1* was present, it was always linked with *gt69-2*, although *gt69-2* loci were identified that lacked linked *rfw-1*. In a sample of *F. oxsporum* isolates, although variation was observed in the number of *gt69-2* and *rfw-1* homologs in these isolates, allelic polymorphisms and discrete haplotypes were not observed (Fig. S6B).

10.1128/mBio.00307-21.6 FIG S6 Phylogenetic distribution of *rfw-1* and *gt69-2* in *Fusarium* species. (A) Phylogenetic tree of *gt69-2* homologs from *Fusarium* species. Predicted amino acid sequences of *gt69-2* from the indicated isolates were used to build a maximum-likelihood phylogenetic tree; accession numbers are provided in Table S3. Results from 100 bootstrap replicates are shown beside each branch. Strains of the same species are shaded with identical colors. Fo, Fusarium oxysporum; Fp, Fusarium proliferatum; Fg, Fusarium graminearum; Ff, Fusarium fujikuroi. Strains with multiple paralogs of *gt69-2* are designated *gt69-2* (I) to *gt69-2* (V), according to the similarity of amino acid sequence to *gt69-2*^FGSC2489^. The *gt69-2* paralogs in the tree are labeled with stars that have linked *rfw-1* alleles; other *gt69-2* alleles indicated in the tree do not have linked *rfw-1* alleles. (B) Genomic situation of the paralogs of *rfw-1* and *gt69*-2 in F. oxysporum strains; accession numbers are provided in Table S3. The *gt69-2* or linked *gt69-2 rfw-1* paralogs in different strains are shown; genes in the same genomic location with the same flanking genes are indicated by identical colors. Arrows with a dotted border indicate that the region does not have an annotated gene, but has similar DNA sequences to the same genomic region of other strains with annotated flanking genes. Download FIG S6, EPS file, 0.5 MB. Copyright © 2021 Li et al. 2021 Li et al. https://creativecommons.org/licenses/by/4.0/ This content is distributed under the terms of the Creative Commons Attribution 4.0 International license.

10.1128/mBio.00307-21.10 Table S3 Accession numbers of RFW-1 and GT69-2 orthologs in different species. Download Table S3, DOCX file, 0.03 MB. Copyright © 2021 Li et al. 2021 Li et al. https://creativecommons.org/licenses/by/4.0/ This content is distributed under the terms of the Creative Commons Attribution 4.0 International license.

In N. crassa, to determine if *gt69-2* plays a role in allorecognition, we cloned the haplogroup II allele of *gt69-2* (haplotype group II isolates lack *rfw-1*) from isolate JW224 (*gt69-2^JW224^*) driven by a *tef-1* promoter, tagged it with GFP, and introduced this construct into the Δ*gt69-2* and Δ*rfw-1*Δ*gt69-2* mutants. The resulting strains Δ*gt69-2* (*gfp*-*gt69-2^JW224^*) and Δ*rfw-1*Δ*gt69-2* (*gfp*-*gt69-2^JW224^*) were used to test the growth and cell fusion phenotype. The Δ*gt69-2* (*gfp*-*gt69-2^JW224^*) and Δ*rfw-1*Δ*gt69-2* (*gfp*-*gt69-2^JW224^*) strains showed similar growth phenotypes to FGSC2489 (Fig. S3E), suggesting that the introduction of *gt69-2^JW224^* into the Δ*gt69-2* mutant restored cell fusion. Consistent with this observation, use of flow cytometry to quantify cell fusion frequencies in pairings between Δ*gt69-2* (*gfp-gt69-2^JW224^*) + *gt69-2^FGSC2489^ rfw-1^FGSC2489^* or Δ*rfw-1*Δ*gt69-2* (*gfp-gt69-2^JW224^*)* + gt69-2^FGSC2489^ rfw-1^FGSC2489^* showed a high frequency of cell fusion (Fig. 7A). Identical to results of pairings between Δ*gt69-2* (*gfp-gt69-2*) + Δ*gt69-2* cells (Fig. 4), the Δ*gt69-2* (*gfp-gt69-2^JW224^*) strain failed to fuse with Δ*gt69-2* cells. We also tested whether coexpression of *gfp*-*gt69-2^JW224^* and *gt69-2^FGSC2489^rfw-1^FGSC2489^* in the same cells would affect growth or cell fusion. However, a *gt69-2^FGSC2489^rfw-1^FGSC2489^* (*gfp-gt69-2^JW224^*) strain showed no obvious defects in growth or cell fusion (Fig. S7). These data indicated that the introduction of the *gt69-2* allele from a different haplogroup was sufficient to complement both the growth and cell fusion defects of the Δ*gt69-2* mutant, but was not sufficient to induce allorecognition and a restriction of cell fusion.

10.1128/mBio.00307-21.7 FIG S7 Phenotypes of strain coexpressing alleles from different *gt69-2* haplogroups. (A) The indicated strains were grown in slant tubes for 7 days. (B) Microscopic assay of cell fusion between FGSC2489 (*gfp-gt69-*2^JW224^) and FM4-64-stained FGSC2489. Download FIG S7, EPS file, 0.3 MB. Copyright © 2021 Li et al. 2021 Li et al. https://creativecommons.org/licenses/by/4.0/ This content is distributed under the terms of the Creative Commons Attribution 4.0 International license.

## DISCUSSION

In this study, we identified a linked gene pair, *gt69-2* and *rfw-1*, that functions to regulate somatic cell fusion in N. crassa. The *gt69-2* locus is predicted to encode a CAP59-like α-1,3-mannosyltransferase and, based on its similarity to C. neoformans *CMT1*, to catalyze the transfer of mannose from GDP-mannose to α-1,3-linked mannose disaccharides (31). A paralog of *CMT1* in C. neoformans, *CAP59*, is required for capsule synthesis by playing a role in the export of the capsular polysaccharide glucuronoxylomannan (31). Both *gt69-2* and *CAP59* orthologs belong to glycosyltransferase family 69 and contain the conserved CAP59 family alpha-1,3-mannosyltransferase catalytic domain. In Aspergillus fumigatus, the Golgi-localized protein ClpA adds an alpha-1-3-linked mannose to glycosylphosphatidylinositol (GPI) anchors (37); *clpA* is a homolog of *Cap59*. GPI anchors are important for anchoring cell surface proteins to the plasma membrane/cell wall (38). The attachment of the GPI anchor occurs in the ER, but the understanding of the maturation of the GPI anchor that occurs in the Golgi is limited.

We hypothesized that GT69-2 functions to modify secreted protein(s), such as GPI-anchored proteins, destined for the cell wall or plasma membrane, or that a small fraction of GT69-2 is trafficked to the cell surface during chemotropic interactions, modifying proteins important for late stages of MAK-2 signaling and cell wall remodeling/dissolution during the process of cell fusion. A wrinkle in this hypothesis was the observation that loss-of-function mutations in *rfw-1* suppressed the cell fusion defect of the Δ*gt69-2* mutant; Δ*gt69-2*Δ*rfw-1* mutants were fusion competent. These data indicated that neither GT69-2 nor RFW-1 are essential for cell fusion in N. crassa, but rather, in the absence of GT69-2, RFW-1 functions to block cell fusion. We predict that in the absence of GT69-2, RFW-1 may inappropriately modify a protein or block secretion of a protein needed for mediating the transition from chemotropic interactions to cell wall dissolution, resulting in the loss of MAK-2 localization at cell contact sites and cessation of the cell fusion process. Localization of MAK-2 to the fusion pore as cell wall dissolution and membrane merger are occurring has been reported previously (20), and MAK-2 kinase activity is required for cell wall dissolution (39).

Consistent with the above hypothesis, overexpression of *rfw-1* resulted in a block in cell fusion, even in the presence of *gt69-2*. The overexpression *rfw-1* strain also showed a conidial separation deficiency associated with an inability to remove cell wall material at the double-doublet stage of conidial development. The phenotype of the *rfw-1* overexpression strain most closely resembles the *csp-2* mutant in N. crassa, where *csp-2 *encodes a homolog of grainy head-like transcription factors (40). An inability to remove the thin connectives between adjacent conidia has been associated with a decrease in autocatalytic activity of the cell wall, hypothesized to be due to a lack of secreted enzymes, such as chitinases (41); a gene encoding a chitinase and additional proteins associated with cell wall structure were identified as transcriptional targets of CSP-2 (40). Two cell wall glycosyl hydrolases, the CGL-1 β-1,3-glucanase and the NAG-1 exochitinase, function in remodeling the cell wall between adjacent conidia to facilitate conidia formation and dissemination (42). Two additional predicted GPI-anchored proteins, BGT-1 and BGT-2, encoding predicted β-1-3 endoglucanases (GH17 family) (43), localize to double-doublets in developing conidia and also to fusion points of germlings and hyphae (44). The Δ*bgt-1* and Δ*bgt-2* mutants display a deficiency in conidial separation, but do not display a cell fusion defect (44). Other mutants in N. crassa that show defects in conidial separation do show defects in cell fusion, however, including loss-of-function mutations in *whi-2*, *csp-6*, and *amph-1* (23, 32). CSP-6 and WHI-2 physically interact (45) and WHI-2, which localizes to the cell periphery, is required for signaling during chemotropic interactions via the MAK-2 MAPK pathway (23). Future studies to identify targets of RFW-1 and GT69-2 should help to understand the molecular basis of the cell wall remodeling process regulated by the RFW-1/GT69-2 system.

In the genomes of *Fusarium* and *Neurospora* species, all predicted *rfw-1* genes were always linked to *gt69-2* genes, although homologs of *gt69-2* occurred without a linked *rfw-1* gene (Fig. S6). These observations suggest that GT69-2 and RFW-1 also function as a pair in species other than in N. crassa. Coevolution of linked genes to maintain physical or functional interactions of their products occurs via coordinated sequence changes between the gene pairs (46). In *Neurospora* species, *gt69-2* orthologs found in two haplogroups showed evidence of balancing selection, similar to other systems regulating allorecognition (25, 27, 29, 30, 47). However, expression of a *gt69-2^JW224^* (haplogroup II allele) in a *gt69-2^FGSC2489^* (haplogroup I allele) strain was insufficient to activate allorecognition and block cell fusion. The *gt69-2^JW224^* allele was fully functional, as it fully complemented the fusion-deficiency phenotype of a Δ*gt69-2* mutant. One possible explanation is that the *gt69-2* alleles from haplogroup II have adapted to the loss of *rfw-1*, while haplogroup I strains need both *gt69-2* and *rfw-1* to correctly modify their targets in the Golgi. Alternatively, it is possible that the evolutionary forces driving balancing selection at *gt69-2/rfw-1* do not reflect the function of these two proteins in cell fusion/conidial separation. Further work to identify the targets of the GT69-2/RFW-1 pair from haplogroup I relative to GT69-2 from haplogroup II will help to resolve this question, in addition to identifying cell membrane/cell wall-associated proteins required for late functions of MAK-2 signaling involved in cell wall dissolution and membrane merger during somatic cell fusion.

## MATERIALS AND METHODS

### Strains and growth conditions.

Standard procedures and protocols for N. crassa can be found on the *Neurospora* homepage at the Fungal Genetics Stock Center (FGSC, www.fgsc.net/Neurospora/NeurosporaProtocolGuide.htm). Vogel’s minimal medium (VMM) (with supplements, if required) was used to culture all strains (48). Crosses were performed on Westergaard’s synthetic crossing medium (49). All the strains used in this study are listed in Table S1 in the supplemental material. The wild N. crassa isolates from a Louisiana population have been previously described (25, 26, 50). FGSC2489 served as the wild-type (WT) control for all experiments and the parental strain for gene engineering, unless stated otherwise.

### Strain construction.

All gene deletion constructs were generated by double-joint PCR (25, 51). The deletion mutants were obtained as described (25, 29). For the Δ*rfw-1*Δ*gt69-2* double mutant, the whole region containing both NCU05915 and NCU05916 was replaced with the hygromycin B-resistance cassette in FGSC2489. For the independently derived ΔNCU05915 Δ*gt69-2* double mutant, *rfw-1* was replaced with the nourseothricin-resistance cassette (52) in the Δ*gt69-2* mutant. Putative deletion mutants were screened for drug resistance and further confirmed by PCR (Fig. S3A and B). The primers are listed in Table S2.

10.1128/mBio.00307-21.9 Table S2 Primers used in this study. Download Table S2, DOCX file, 0.02 MB. Copyright © 2021 Li et al. 2021 Li et al. https://creativecommons.org/licenses/by/4.0/ This content is distributed under the terms of the Creative Commons Attribution 4.0 International license.

To generate the Δ*gt69-2* *gfp-gt69-2* strain, *superfoldergfp*-fused *gt69-2* was cloned into a pMF272-derived vector to create *gfp* fusions (25) using HiFi DNA assembly (New England BioLabs) under the regulation of the *ccg-1* promoter (53), and introduced in the *his-3* locus (25, 54) of a Δ*gt69-2* strain. Positive transformants were backcrossed to a Δ*gt69-2* mutant of the opposite mating type to obtain homokaryotic strains that were subsequently confirmed by PCR (Fig. S3A and B). Similar approaches were used to generate Δ*gt69-2* (*gfp*-*gt69-2^JW224^*), Δ*rfw-1* (*gfp*-*rfw-1*), and Δ*rfw-1*Δ*gt69-2* (*gfp*-*rfw-1*) strains.

The FGSC2489^sec-9swap^ strain, which was engineered to carry *sec-*9^GRD2^ at the native *sec-9* locus, has been previously described (30). The Δ*rfw-1* and/or Δ*gt69-2* mutants were crossed with FGSC2489^sec-9swap^ to obtain the resulting sec-9swap strains.

### Bulk segregant analysis.

Bulk segregant analysis (BSA) followed by whole-genome resequencing was performed as previously described (25). Approximately 60 ng of genomic DNA from ∼49 progeny strains in each DNA pool was used for library preparation and sequencing. All paired-end libraries were sequenced on a HiSeq2000 sequencing platform using standard Illumina operating procedures (QB3 Genomics Lab, University of California, Berkeley).

### Microscopy.

Cell fusion experiments were performed as described (25). Cytoplasmic or histone 1-tagged GFP-expressing cells and FM-64-stained (Thermo Fisher Scientific) cells were mixed in a 1:1 proportion and incubated on VMM plates at 30°C in the dark for 4 h. Cytoplasmic mixing was examined with a Zeiss Axioskop 2 microscope equipped with a Q Imaging Retiga-2000R camera (Surrey) using a 40×/1.30 Plan-Neofluar oil immersion objective and the iVision Mac 4.5 software.

Heterokaryotic strains bearing both GFP and mCherry fluorescent proteins were prepared as described (25) for colocalization analysis. Images were taken with a Leica SD6000 confocal microscope equipped with a Yokogawa CSU-X1 spinning disk head, and a 488-nm or 561-nm laser controlled by Metamorph software.

For MAK-2 oscillation experiments, conidia from strains expressing MAK-2-GFP were prepared for microscopy as described (25). Time-lapse microscopy was performed using the confocal microscope system as described above. Images were captured at 30 s intervals. The software ImageJ was used for image processing. Fluorescence signals were quantified as previously described (20).

### Transmission electron microscopy.

Conidia were inoculated in 100 ml of liquid VMM at a final concentration of 10^6^ conidia/ml for 5 hat 30°C (shaking at 220 rpm for 2.5 h and standing for 2.5 h). Cells were harvested by centrifugation and then fixed with electron microscopy fix buffer (2% glutaraldehyde, 4% paraformaldehyde, 0.04 M phosphate buffer [pH 7.0]), followed by 2% KMnO_4_ treatment. Samples were dehydrated using a graded ethanol series before embedding the samples in resin.

### Flow cytometry.

Flow cytometry was performed as described (29). For each experiment, 20,000 events per sample were recorded on a BD LSR Fortessa X-20 (BD Biosciences, Franklin Lakes, NJ, USA). Cell death frequencies were analyzed with a specifically designed MATLAB script (29). Each experiment was performed at least three times.

### Growth assays.

To evaluate growth rate, a hyphal plug (1 mm^2^) or 5 μl of a conidial suspension (10^6^ conidia/ml) was inoculated onto the center of 14.2-cm diameter petri dishes and grown at 30°C in constant dark. The colony diameter was recorded twice a day.

Cell wall stress assays were conducted on VMM + FGS with 1.3 μg/ml caspofungin, 1.5 mg/ml calcofluor white, or 1 mg/ml Congo red as described (55). A 1:5 dilution series was prepared starting with a concentration of 10^6^ conidia/ml. Conidial solutions were then spotted onto freshly poured plates at 5 μl per spot.

### Phylogenetic analysis.

The sequences of *gt69-2* and *rfw-1* orthologs were obtained by a BLAST search using NCU05915 and NCU05916 from FGSC2489 as a query against sequence database of *Neurospora* (26, 56–58) and *Fusarium* (http://fungi.ensembl.org/index.html) species. Amino acid alignments were carried out using MAFFT alignments (59) and phylogenetic trees were constructed using MEGAX (60). Tajima’s D tests were processed using DnaSP6 (61).

## References

[1] Hickey PC, Jacobson D, Read ND, Glass NL. 2002. Live-cell imaging of vegetative hyphal fusion in *Neurospora crassa*. Fungal Genet Biol 37:109–119. doi:10.1016/s1087-1845(02)00035-x. 12223195

[2] Pieuchot L, Lai J, Loh RA, Leong FY, Chiam KH, Stajich J, Jedd G. 2015. Cellular subcompartments through cytoplasmic streaming.Dev Cell 34:410–420. doi:10.1016/j.devcel.2015.07.017. 26305593

[3] Simonin A, Palma-Guerrero J, Fricker M, Glass NL. 2012. Physiological significance of network organization in fungi. Eukaryot Cell 11:1345–1352. doi:10.1128/EC.00213-12. 22962278 PMC3486018

[4] Roper M, Simonin A, Hickey PC, Leeder A, Glass NL. 2013. Nuclear dynamics in a fungal chimera. Proc Natl AcadSci U S A 110:12875–12880. doi:10.1073/pnas.1220842110. PMC3740868 23861490

[5] Mela AP, Rico-Ramirez AM, Glass NL. 2020. Syncytia in fungi. Cells 9:2255. doi:10.3390/cells9102255. PMC7600787 33050028

[6] Strom NB, Bushley KE. 2016. Two genomes are better than one: history, genetics, and biotechnological applications of fungal heterokaryons. Fungal BiolBiotechnol 3:4. doi:10.1186/s40694-016-0022-x. PMC5611628 28955463

[7] Bastiaans E, Debets AJ, Aanen DK. 2015. Experimental demonstration of the benefits of somatic fusion and the consequences for allorecognition.Evolution 69:1091–1099. doi:10.1111/evo.12626. 25688421

[8] Craven KD, Velez H, Cho Y, Lawrence CB, Mitchell TK. 2008. Anastomosis is required for virulence of the fungal necrotroph*Alternariabrassicicola*. Eukaryot Cell 7:675–683. doi:10.1128/EC.00423-07. 18310356 PMC2292617

[9] Charlton ND, Shoji JY, Ghimire SR, Nakashima J, Craven KD. 2012. Deletion of the fungal gene *soft* disrupts mutualistic symbiosis between the grass endophyte *Epichloefestucae* and the host plant. Eukaryot Cell 11:1463–1471. doi:10.1128/EC.00191-12. 23042130 PMC3536286

[10] Mehrabi R, Bahkali AH, Abd-Elsalam KA, Moslem M, Ben M'barek S, Gohari AM, Jashni MK, Stergiopoulos I, Kema GH, de Wit PJ. 2011. Horizontal gene and chromosome transfer in plant pathogenic fungi affecting host range. FEMS Microbiol Rev 35:542–554. doi:10.1111/j.1574-6976.2010.00263.x. 21223323

[11] Czaran T, Hoekstra RF, Aanen DK. 2014. Selection against somatic parasitism can maintain allorecognition in fungi. Fungal Genet Biol 73:128–137. doi:10.1016/j.fgb.2014.09.010. 25305337

[12] Biella S, Smith ML, Aist JR, Cortesi P, Milgroom MG. 2002. Programmed cell death correlates with virus transmission in a filamentous fungus. Proc BiolSci 269:2269–2276. doi:10.1098/rspb.2002.2148. PMC1691157 12455515

[13] Daskalov A, Heller J, Herzog S, Fleissner A, Glass NL. 2017. Molecular mechanisms regulating cell fusion and heterokaryon formation in filamentous fungi. MicrobiolSpectr 5. doi:10.1128/microbiolspec.FUNK-0015-2016. PMC11687462 28256191

[14] Goncalves AP, Heller J, Daskalov A, Videira A, Glass NL. 2017. Regulated forms of cell death in fungi. Front Microbiol 8:1837. doi:10.3389/fmicb.2017.01837. 28983298 PMC5613156

[15] Saupe SJ. 2000. Molecular genetics of heterokaryon incompatibility in filamentous ascomycetes. MicrobiolMolBiol Rev 64:489–502. doi:10.1128/mmbr.64.3.489-502.2000. PMC99001 10974123

[16] Goncalves AP, Glass NL. 2020. Fungal social barriers: to fuse, or not to fuse, that is the question. CommunIntegrBiol 13:39–42. doi:10.1080/19420889.2020.1740554. PMC7159315 32313605

[17] Goncalves AP, Heller J, Rico-Ramirez AM, Daskalov A, Rosenfield G, Glass NL. 2020. Conflict, competition, and cooperation regulate social interactions in filamentous fungi. Annu Rev Microbiol 74:693–712. doi:10.1146/annurev-micro-012420-080905. 32689913

[18] Fischer MS, Glass NL. 2019. Communicate and fuse: how filamentous fungi establish and maintain an interconnected mycelial network. Front Microbiol 10:619. doi:10.3389/fmicb.2019.00619. 31001214 PMC6455062

[19] Roca MG, Arlt J, Jeffree CE, Read ND. 2005. Cell biology of conidial anastomosis tubes in *Neurospora crassa*. Eukaryot Cell 4:911–919. doi:10.1128/EC.4.5.911-919.2005. 15879525 PMC1140100

[20] Fleissner A, Leeder AC, Roca MG, Read ND, Glass NL. 2009. Oscillatory recruitment of signaling proteins to cell tips promotes coordinated behavior during cell fusion. Proc Natl AcadSci U S A 106:19387–19392. doi:10.1073/pnas.0907039106. PMC2780775 19884508

[21] Jonkers W, Fischer MS, Do HP, Starr TL, Glass NL. 2016. Chemotropism and cell fusion in *Neurospora crassa* relies on the formation of distinct protein complexes by HAM-5 and a novel protein HAM-14. Genetics 203:319–334. doi:10.1534/genetics.115.185348. 27029735 PMC4858782

[22] Dettmann A, Heilig Y, Valerius O, Ludwig S, Seiler S. 2014. Fungal communication requires the MAK-2 pathway elements STE-20 and RAS-2, the NRC-1 adapter STE-50 and the MAP kinase scaffold HAM-5. PLoS Genet 10:e1004762. doi:10.1371/journal.pgen.1004762. 25411845 PMC4239118

[23] Goncalves AP, Chow KM, Cea-Sanchez S, Glass NL. 2019. WHI-2 regulates intercellular communication via a MAP kinase signaling complex. Front Microbiol 10:3162. doi:10.3389/fmicb.2019.03162. 32038591 PMC6987382

[24] Herzog S, Schumann MR, Fleißner A. 2015. Cell fusion in *Neurospora crassa*.CurrOpinMicrobiol 28:53–59. doi:10.1016/j.mib.2015.08.002. 26340439

[25] Heller J, Zhao J, Rosenfield G, Kowbel DJ, Gladieux P, Glass NL. 2016. Characterization of greenbeard genes involved in long-distance kind discrimination in a microbial eukaryote. PLoSBiol 14:e1002431. doi:10.1371/journal.pbio.1002431. PMC4831770 27077707

[26] Zhao J, Gladieux P, Hutchison E, Bueche J, Hall C, Perraudeau F, Glass NL. 2015. Identification of allorecognition loci in *Neurospora crassa* by genomics and evolutionary approaches. MolBiolEvol 32:2417–2432. doi:10.1093/molbev/msv125. PMC4540973 26025978

[27] Daskalov A, Gladieux P, Heller J, Glass NL. 2019. Programmed cell death in *Neurospora crassa* is controlled by the allorecognition determinant *rcd-1*. Genetics 213:1387–1400. doi:10.1534/genetics.119.302617. 31636083 PMC6893366

[28] Daskalov A, Mitchell PS, Sandstrom A, Vance RE, Glass NL. 2020. Molecular characterization of a fungal gasdermin-like protein. Proc Natl AcadSci U S A 117:18600–18607. doi:10.1073/pnas.2004876117. PMC7414189 32703806

[29] Goncalves AP, Heller J, Span EA, Rosenfield G, Do HP, Palma-Guerrero J, Requena N, Marletta MA, Glass NL. 2019. Allorecognition upon fungal cell-cell contact determines social cooperation and impacts the acquisition of multicellularity. CurrBiol 29:3006–3017. doi:10.1016/j.cub.2019.07.060. 31474536

[30] Heller J, Clave C, Gladieux P, Saupe SJ, Glass NL. 2018. NLR surveillance of essential SEC-9 SNARE proteins induces programmed cell death upon allorecognition in filamentous fungi. Proc Natl AcadSci U S A 115:E2292–E2301. doi:10.1073/pnas.1719705115. PMC5878007 29463729

[31] Sommer U, Liu H, Doering TL. 2003. An alpha-1,3-mannosyltransferase of *Cryptococcus neoformans*. J BiolChem 278:47724–47730. doi:10.1074/jbc.M307223200. 14504286

[32] Fu C, Iyer P, Herkal A, Abdullah J, Stout A, Free SJ. 2011. Identification and characterization of genes required for cell-to-cell fusion in *Neurospora crassa*. Eukaryot Cell 10:1100–1109. doi:10.1128/EC.05003-11. 21666072 PMC3165452

[33] Palma-Guerrero J, Leeder AC, Welch J, Glass NL. 2014. Identification and characterization of LFD1, a novel protein involved in membrane merger during cell fusion in *Neurospora crassa*. MolMicrobiol 92:164–182. doi:10.1111/mmi.12545. 24673848

[34] Afzali B, Lombardi G, Lechler RI. 2008. Pathways of major histocompatibility complex allorecognition. CurrOpin Organ Transplant 13:438–444. doi:10.1097/MOT.0b013e328309ee31. PMC3815495 18685342

[35] Nydam ML, De Tomaso AW. 2012. The fester locus in *Botryllusschlosseri* experiences selection. BMC EvolBiol 12:249. doi:10.1186/1471-2148-12-249. PMC3549757 23259925

[36] Blackstone NW. 2020. Evolutionary conflict and coloniality in animals.J ExpZool B Mol Dev Evol doi:10.1002/jez.b.22924. 31922350

[37] Kruger AT, Engel J, Buettner FF, Routier FH. 2016. *Aspergillus fumigatus* Cap59-like protein A is involved in alpha1,3-mannosylation of GPI-anchors. Glycobiol 26:30–38. doi:10.1093/glycob/cwv078. 26369907

[38] Orlean P, Menon AK. 2007. Thematic review series: lipid posttranslational modifications. GPI anchoring of protein in yeast and mammalian cells, or: how we learned to stop worrying and love glycophospholipids. J Lipid Res 48:993–1011. doi:10.1194/jlr.R700002-JLR200. 17361015

[39] Serrano A, Illgen J, Brandt U, Thieme N, Letz A, Lichius A, Read ND, Fleißner A. 2018. Spatio-temporal MAPK dynamics mediate cell behavior coordination during fungal somatic cell fusion. J Cell Sci 131:jcs213462. doi:10.1242/jcs.213462. 29592970 PMC5992588

[40] Pare A, Kim M, Juarez MT, Brody S, McGinnis W. 2012. The functions of grainy head-like proteins in animals and fungi and the evolution of apical extracellular barriers.PLoS One 7:e36254. doi:10.1371/journal.pone.0036254. 22590528 PMC3348937

[41] Selitrennikoff CP, Nelson RE, Siegel RW. 1974. Phase-specific genes for macroconidiation in *Neurospora crassa*. Genetics 78:679–690. doi:10.1093/genetics/78.2.679. 4280981 PMC1213227

[42] Ao J, Aldabbous M, Notaro MJ, Lojacono M, Free SJ. 2016. A proteomic and genetic analysis of the *Neurospora crassa* conidia cell wall proteins identifies two glycosyl hydrolases involved in cell wall remodeling. Fungal Genet Biol 94:47–53. doi:10.1016/j.fgb.2016.07.003. 27381444 PMC4972661

[43] Lombard V, GolacondaRamulu H, Drula E, Coutinho PM, Henrissat B. 2014. The carbohydrate-active enzymes database (CAZy) in 2013. Nucleic Acids Res 42:D490–5. doi:10.1093/nar/gkt1178. 24270786 PMC3965031

[44] Martinez-Nunez L, Riquelme M. 2015. Role of BGT-1 and BGT-2, two predicted GPI-anchored glycoside hydrolases/glycosyltransferases, in cell wall remodeling in *Neurospora crassa*. Fungal Genet Biol 85:58–70. doi:10.1016/j.fgb.2015.11.001. 26541633

[45] Zhou X, Wang B, Emerson JM, Ringelberg CS, Gerber SA, Loros JJ, Dunlap JC. 2018. A HAD family phosphatase CSP-6 regulates the circadian output pathway in *Neurospora crassa*. PLoS Genet 14:e1007192. doi:10.1371/journal.pgen.1007192. 29351294 PMC5800702

[46] de Juan D, Pazos F, Valencia A. 2013. Emerging methods in protein co-evolution.Nat Rev Genet 14:249–261. doi:10.1038/nrg3414. 23458856

[47] Wu J, Saupe SJ, Glass NL. 1998. Evidence for balancing selection operating at the *het-c*heterokaryon incompatibility locus in a group of filamentous fungi. Proc NatlAcadSciU S A 95:12398–12403. doi:10.1073/pnas.95.21.12398. PMC22843 9770498

[48] Vogel HJ. 1956. A convenient growth medium for Neurospora (Medium N). Microb Genet Bull 13:42–43.

[49] Westergaard M, Mitchell HK. 1947. Neurospora V. A synthetic medium favoring sexual reproduction.Am J Bot 34:573–577. doi:10.2307/2437339.

[50] Palma-Guerrero J, Hall CR, Kowbel D, Welch J, Taylor JW, Brem RB, Glass NL. 2013. Genome wide association identifies novel loci involved in fungal communication. PLoS Genet 9:e1003669. doi:10.1371/journal.pgen.1003669. 23935534 PMC3731230

[51] Yu JH, Hamari Z, Han KH, Seo JA, Reyes-Dominguez Y, Scazzocchio C. 2004. Double-joint PCR: a PCR-based molecular tool for gene manipulations in filamentous fungi. Fungal Genet Biol 41:973–981. doi:10.1016/j.fgb.2004.08.001. 15465386

[52] Alshahni MM, Makimura K, Yamada T, Takatori K, Sawada T. 2010. Nourseothricin acetyltransferase: a new dominant selectable marker for the dermatophyte *Trichophyton mentagrophytes*. Med Mycol 48:665–668. doi:10.3109/13693780903330555. 19886766

[53] Freitag M, Hickey PC, Raju NB, Selker EU, Read ND. 2004. GFP as a tool to analyze the organization, dynamics and function of nuclei and microtubules in *Neurospora crassa*. Fungal Genet Biol 41:897–910. doi:10.1016/j.fgb.2004.06.008. 15341912

[54] Margolin BS, Freitag M, Selker EU. 1997. Improved plasmids for gene targeting at the *his-3* locus of *Neurospora crassa* by electroporation. Fungal Genet Newslett 44:34–36. doi:10.4148/1941-4765.1281.

[55] Fischer MS, Wu VW, Lee JE, O'Malley RC, Glass NL. 2018. Regulation of cell-to-cell communication and cell wall integrity by a network of MAP kinase pathways and transcription factors in *Neurospora crassa*. Genetics 209:489–506. doi:10.1534/genetics.118.300904. 29678830 PMC5972422

[56] Gladieux P, Wilson BA, Perraudeau F, Montoya LA, Kowbel D, Hann-Soden C, Fischer M, Sylvain I, Jacobson DJ, Taylor JW. 2015. Genomic sequencing reveals historical, demographic and selective factors associated with the diversification of the fire-associated fungus *Neurospora discreta*. MolEcol 24:5657–5675. doi:10.1111/mec.13417. 26453896

[57] Corcoran P, Anderson JL, Jacobson DJ, Sun Y, Ni P, Lascoux M, Johannesson H. 2016. Introgression maintains the genetic integrity of the mating-type determining chromosome of the fungus *Neurospora tetrasperma*. Genome Res 26:486–498. doi:10.1101/gr.197244.115. 26893460 PMC4817772

[58] Sun Y, Svedberg J, Hiltunen M, Corcoran P, Johannesson H. 2017. Large-scale suppression of recombination predates genomic rearrangements in *Neurospora tetrasperma*. Nat Commun 8:1140. doi:10.1038/s41467-017-01317-6. 29074958 PMC5658415

[59] Katoh K, Standley DM. 2013. MAFFT multiple sequence alignment software version 7: improvements in performance and usability. MolBiolEvol 30:772–780. doi:10.1093/molbev/mst010. PMC3603318 23329690

[60] Kumar S, Stecher G, Li M, Knyaz C, Tamura K. 2018. MEGA X: molecular evolutionary genetics analysis across computing platforms. MolBiolEvol 35:1547–1549. doi:10.1093/molbev/msy096. PMC5967553 29722887

[61] Rozas J, Ferrer-Mata A, Sanchez-DelBarrio JC, Guirao-Rico S, Librado P, Ramos-Onsins SE, Sanchez-Gracia A. 2017. DnaSP 6: DNA sequence polymorphism analysis of large data sets. MolBiolEvol 34:3299–3302. doi:10.1093/molbev/msx248. 29029172

