# Supplementary material for: The Predicted Mannosyltransferase GT69-2 Antagonizes RFW-1 To Regulate Cell Fusion in Neurospora crassa
Source: mBio. 2021 Mar 16;12(2):e00307-21. doi: 10.1128/mBio.00307-21 (PMC8092235; doi:10.1128/mBio.00307-21)
Supplement: Table S1 [file mBio.00307-21-st001.docx]

**Table S1. Strains of *Neurospora crassa* used in this study**

| Strain | Genotype description | Source |
| --- | --- | --- |
| FGSC2489 (74-OR23-IV) | Wild type | FGSC |
| FGSC7918 | ∆*mus-51*::*bar+* | FGSC |
| FGSC2489/GFP | *his-3*::*Pccg-1-gfp* | FGSC |
| Δ*gt69-2* | *∆NCU05916::hygR* | This study |
| *Δrfw-1* | *∆NCU05915::hygR* | This study |
| Δ*gt69-2*Δ*rfw-1* | *∆NCU05915∆NCU05916::hygR* | This study |
| FGSC2489 (MAK-2-GFP) | *his-3::Pccg-1- mak-2-gfp* | (1) |
| Δ*rfw-1*, GFP | *∆NCU05915::hygR his-3::Pccg-1-gfp* | This study |
| Δ*gt69-2*, GFP | *∆NCU05916::hygR his-3::Pccg-1-gfp* | This study |
| Δ*gt69-2*Δ*rfw-1*, GFP | *∆NCU05915∆NCU05916::hygR his-3::Pccg-1-gfp* | This study |
| Δ*rfw-1* (MAK-2-GFP) | *∆NCU05915::hygR his-3::Pccg-1-mak-2-gfp* | This study |
| Δ*gt69-2* (MAK-2-GFP) | *∆NCU05916::hygR his-3::Pccg-1- mak-2-gfp* | This study |
| Δ*gt69-2*Δ*rfw-1* (MAK-2-GFP) | *∆NCU05915∆NCU05916::hygR his-3::Pccg-1- mak-2-gfp* | This study |
| ΔΔ*sec-9* (*sec-9* swap) | *sec-9^JW199^::sec-9 Δplp-1Δplp-2::hygR* | (2) |
| Δ*gt69-2*^sec-9swap^ | *∆NCU05916::hygR sec-9^JW199^::sec-9 Δplp-1Δplp-2::hygR* | This study |
| Δ*rfw-1*^sec-9swap^ | *∆NCU05915::hygR sec-9^JW199^::sec-9 Δplp-1Δplp-2::hygR* | This study |
| Δgt69-2Δrfw-1^sec-9swap^ | *∆NCU05915∆NCU05916::hygR sec-9^JW199^::sec-9 Δplp-1Δplp-2::hygR* | This study |
| ΔNCU05915Δ*gt69-2* | *∆NCU05915::nat+ ∆NCU05916::hygR* | This study |
| Δ*gt69-2* (GFP-GT69-2) | *∆NCU05916::hygR his-3::Pccg-1-gfp-NCU05916* | This study |
| Δ*rfw-1* (GFP-RFW-1) | *∆NCU05915::hygR his-3::Pccg-1-gfp-NCU05915* | This study |
| Δ*gt69-2*Δ*rfw-1* (GFP- RFW-1) | *∆NCU05915 ∆NCU05916::hygR his-3::Pccg-1-gfp-NCU05915* | This study |
| Δ*gt69-2* (GFP- GT69-2^JW224^) | *∆NCU05916::hygR his-3::Pccg-1-gfp-NCU05916^JW224^* | This study |
| Δ*gt69-2*Δ*rfw-1* (GFP- GT69-2^JW224^) | *∆NCU05915 ∆NCU05916::hygR his-3::Pccg-1-gfp- NCU05916^JW224^* | This study |
| ERV25-mCherry | *erv-25:: hygR csr+1::Pgpd-erv-25-mCherry-6×His* | (3) |
| RFP-VPS-52 | *his-3::Pccg1-rfp-vps-52* | (3) |

**References**

1. Fleissner A, Leeder AC, Roca MG, Read ND, Glass NL. 2009. Oscillatory recruitment of signaling proteins to cell tips promotes coordinated behavior during cell fusion. Proc Natl Acad Sci U S A 106:19387-92.

2. Heller J, Clave C, Gladieux P, Saupe SJ, Glass NL. 2018. NLR surveillance of essential SEC-9 SNARE proteins induces programmed cell death upon allorecognition in filamentous fungi. Proc Natl Acad Sci U S A 115:E2292-E2301.

3. Palma-Guerrero J, Zhao J, Goncalves AP, Starr TL, Glass NL. 2015. Identification and characterization of LFD-2, a predicted fringe protein required for membrane integrity during cell fusion in *Neurospora crassa*. Eukaryot Cell 14:265-77.
