# Supplementary material for: The Predicted Mannosyltransferase GT69-2 Antagonizes RFW-1 To Regulate Cell Fusion in Neurospora crassa
Source: mBio. 2021 Mar 16;12(2):e00307-21. doi: 10.1128/mBio.00307-21 (PMC8092235; doi:10.1128/mBio.00307-21)
Supplement: Table S3 [file mBio.00307-21-st003.docx]

**Table S3 Accession numbers of RFW-1 and GT69-2 orthologs in different species**

| **Species** | **f. sp.** | **Strain name** | **RFW-1 ortholog** | **GT69-2 ortholog** | **Note** |
| --- | --- | --- | --- | --- | --- |
| *Neurospora crassa* |  | FGSC2489 | XP_958115.3 | XP_958227.1 |  |
| *Neurospora crassa* |  | JW148 | (1, 2) | |  |
| *Neurospora crassa* |  | JW196 |  |  |  |
| *Neurospora crassa* |  | JW258 |  |  |  |
| *Neurospora crassa* |  | JW193 |  |  |  |
| *Neurospora crassa* |  | JW220 |  |  |  |
| *Neurospora crassa* |  | JW228 |  |  |  |
| *Neurospora crassa* |  | JW242 |  |  |  |
| *Neurospora crassa* |  | P4476 |  |  |  |
| *Neurospora crassa* |  | P4463 |  |  |  |
| *Neurospora crassa* |  | P4452 |  |  |  |
| *Neurospora crassa* |  | JW246 |  |  |  |
| *Neurospora crassa* |  | JW22 |  |  |  |
| *Neurospora crassa* |  | JW179 |  |  |  |
| *Neurospora crassa* |  | JW204 |  |  |  |
| *Neurospora crassa* |  | JW222 |  |  |  |
| *Neurospora crassa* |  | JW224 |  |  |  |
| *Neurospora crassa* |  | P4468 |  |  |  |
| *Neurospora crassa* |  | JW75 |  |  |  |
| *Neurospora crassa* |  | JW199 |  |  |  |
| *Neurospora crassa* |  | P4479 |  |  |  |
| *Neurospora crassa* |  | P4489 |  |  |  |
| *Neurospora crassa* |  | P4471 |  |  |  |
| *Neurospora discreta* |  | AKFA2 |  |  |  |
| *Neurospora discreta* |  | AKFA6 |  |  |  |
| *Neurospora discreta* |  | AKFA12 |  |  |  |
| *Neurospora discreta* |  | AKFA16 |  |  |  |
| *Neurospora discreta* |  | AKFA31 |  |  |  |
| *Neurospora discreta* |  | AKFK20 |  |  |  |
| *Neurospora discreta* |  | CAW973 |  |  |  |
| *Neurospora discreta* |  | CAW967 |  |  |  |
| *Neurospora discreta* |  | NMBE781 |  |  |  |
| *Neurospora tetrasperma* |  | FGSC2508 | XP_009854682.1 | XP_009854681.1 |  |
| *Neurospora tetrasperma* |  | FGSC2509 | EGZ67760.1 | EGZ67762.1 |  |
| *Neurospora tetrasperma* |  | 965a | (3, 4) | |  |
| *Neurospora tetrasperma* |  | CJ01 |  |  |  |
| *Neurospora tetrasperma* |  | CJ02 |  |  |  |
| *Neurospora tetrasperma* |  | CJ03 |  |  |  |
| *Neurospora tetrasperma* |  | CJ04 |  |  |  |
| *Neurospora tetrasperma* |  | CJ05 |  |  |  |
| *Neurospora tetrasperma* |  | CJ06 |  |  |  |
| *Neurospora tetrasperma* |  | CJ07 |  |  |  |
| *Neurospora tetrasperma* |  | CJ08 |  |  |  |
| *Neurospora tetrasperma* |  | CJ09 |  |  |  |
| *Neurospora tetrasperma* |  | CJ73 |  |  |  |
| *Neurospora tetrasperma* |  | CJ85 |  |  |  |
| *Fusarium oxysporum* | f. sp. *cubense* race 4 | race 4 | EMT65851.1 | EMT65850.1 | Paralog I |
|  |  |  |  | EMT67848.1 | Paralog III |
| *Fusarium oxysporum* | f. sp. *cubense* tropical race 4 | 54006 | XP_031067586.1 | XP_031067584.1 | Paralog I |
|  |  |  | XP_031057665.1 | XP_031057666.1 | Paralog III |
| *Fusarium oxysporum* | f. sp. *melonis* | 26406 | EXK29963.1 | EXK29960.1 | Paralog I |
|  |  |  | EXK31309.1 | EXK31308.1 | Paralog V |
|  |  |  |  | EXK25853.1 | Paralog III |
|  |  |  |  | EXK24300.1 | Paralog IV |
| *Fusarium oxysporum* | f. sp. l*ycopersici* | 4287 | XP_018249887.1 | XP_018249888.1 | Paralog I |
|  |  |  | XP_018237810.1 | XP_018237809.1 | Paralog V |
|  |  |  |  | XP_018258428.1 | Paralog III |
| *Fusarium oxysporum* | f. sp. *conglutinans* race 2 | 54008 | EXL76420.1 | EXL76421.1 | Paralog I |
|  |  |  | EXL83904.1 | EXL83903.1 | Paralog V |
|  |  |  |  | EXL65545.1 | Paralog III |
| *Fusarium oxysporum* | f. sp. *vasinfectum* | 25433 | EXM27031.1 | EXM27029.1 | Paralog I |
|  |  |  | EXM23473.1 | EXM23474.1 | Paralog V |
|  |  |  |  | EXM20780.1 | Paralog III |
| *Fusarium oxysporum* |  | Fo47 | EWZ35888.1 | EWZ35889.1 | Paralog I |
|  |  |  | EWZ39290.1 | EWZ39291.1 | Paralog V |
|  |  |  |  | EWZ31405.1 | Paralog III |
| *Fusarium oxysporum* | f. sp. *radicis-lycopersici* | 26381 | EXL55431.1 | EXL55432.1 | Paralog I |
|  |  |  | EXL50844.1 | EXL50845.1 | Paralog V |
|  |  |  |  | EXL52732.1 | Paralog III |
| *Fusarium oxysporum* | f. sp. *radicis-cucumerinum* | Forc016 | PCD29318.1 | PCD29319.1 | Paralog I |
|  |  |  |  | PCD24533.1 | Paralog III |
|  |  |  | PCD33389.1 | PCD33388.1 | Paralog V |
|  |  |  |  | PCD22052.1 | Paralog II |
|  |  |  |  | PCD22087.1 | Paralog IV |
| *Fusarium oxysporum* | f. sp. l*ycopersici* | MN25 | EWZ96113.1 | EWZ96110.1 | Paralog I |
|  |  |  | EWZ83751.1 | EWZ83750.1 | Paralog V |
|  |  |  |  | EWZ80783.1 | Paralog III |
| *Fusarium oxysporum* | f. sp. *cubense* race 1 | race 1 | ENH75030 | ENH75029.1 | Paralog I |
|  |  |  | ENH71030.1 | ENH71029.1 | Paralog V |
|  |  |  |  | ENH72054.1 | Paralog III |
| *Fusarium oxysporum* |  | V64-1 | SCO89753.1 | SCO89754.1 | Paralog I |
|  |  |  | SCO87136.1 | SCO87135.1 | Paralog V |
|  |  |  |  | SCO91275.1 | Paralog III |
|  |  |  |  | SCO92527.1 | Paralog IV |
| *Fusarium oxysporum* |  | NRRL 32931 | XP_031034355.1 | XP_031034356.1 | Paralog I |
|  |  |  | XP_031038094.1 | XP_031038093.1 | Paralog V |
|  |  |  |  | XP_031030905.1 | Paralog III |
| *Fusarium oxysporum* | f. sp. *pisi* | HDV247 | EXA38020.1 | EXA38021.1 | Paralog I |
|  |  |  | EXA40099.1 | EXA40100.1 | Paralog V |
|  |  |  |  | EXA30396.1 | Paralog III |
| *Fusarium oxysporum* | f. sp. *raphani* | 54005 | EXK89685.1 | EXK89683.1 | Paralog I |
|  |  |  | EXK98223.1 | EXK98224.1 | Paralog II |
| *Fusarium fujikuroi* |  | E282 | SCO06036.1 | SCO06038.1 | Paralog I |
|  |  |  | SCN83425.1 | SCN83422.1 | Paralog V |
| *Fusarium fujikuroi* |  | KSU 3368 | KLP21434.1 | KLP21435.1 | Paralog I |
|  |  |  |  | KLO89248.1 | Paralog V |
| *Fusarium fujikuroi* |  | FSU48 | SCV52589.1 | SCV52587.1 | Paralog I |
|  |  |  | SCV36549.1 | SCV36546.1 | Paralog V |
| *Fusarium fujikuroi* |  | IMI 58289 | XP_023435770.1 | XP_023436161.1 | Paralog I |
|  |  |  | XP_023431894.1 | XP_023431895.1 | Paralog V |
| *Fusarium fujikuroi* |  | FGSC 8932 | KLP07881.1 | KLP07880.1 | Paralog I |
|  |  |  | KLP11323.1 | KLP11324.1 | Paralog V |
| *Fusarium fujikuroi* |  | KSU X-10626 | KLO79659.1 | KLO79660.1 | Paralog I |
|  |  |  | KLO84486.1 | KLO84485.1 | Paralog V |
| *Fusarium fujikuroi* |  | B14 | SCV44260.1 | SCV44263.1 | Paralog I |
|  |  |  | SCV34746.1 | SCV34747.1 | Paralog V |
| *Fusarium proliferatum* |  | ET1 | XP_031088212.1 | XP_031088213.1 | Paralog I |
|  |  |  | XP_031082502.1 | XP_031082503.1 | Paralog V |
| *Fusarium proliferatum* |  | NRRL62905 | CVL10635.1 | CVL10634.1 | Paralog I |
|  |  |  | CVL10851.1 | CVL10850.1 | Paralog V |
| *Fusarium graminearum* |  | PH-1 | SCB65895.1 | SCB65894.1 |  |
| *Fusarium graminearum* |  | CS3005 | EYB32315.1 | EYB32458.1 |  |

**References**

1. Heller J, Zhao J, Rosenfield G, Kowbel DJ, Gladieux P, Glass NL. 2016. Characterization of Greenbeard Genes Involved in Long-Distance Kind Discrimination in a Microbial Eukaryote. PLoS Biol 14:e1002431.

2. Heller J, Clave C, Gladieux P, Saupe SJ, Glass NL. 2018. NLR surveillance of essential SEC-9 SNARE proteins induces programmed cell death upon allorecognition in filamentous fungi. Proc Natl Acad Sci U S A 115:E2292-E2301.

3. Corcoran P, Anderson JL, Jacobson DJ, Sun Y, Ni P, Lascoux M, Johannesson H. 2016. Introgression maintains the genetic integrity of the mating-type determining chromosome of the fungus *Neurospora tetrasperma*. Genome Res 26:486-98.

4. Sun Y, Svedberg J, Hiltunen M, Corcoran P, Johannesson H. 2017. Large-scale suppression of recombination predates genomic rearrangements in *Neurospora tetrasperma*. Nat Commun 8:1140.
