# Supplementary material for: The Predicted Mannosyltransferase GT69-2 Antagonizes RFW-1 To Regulate Cell Fusion in Neurospora crassa
Source: mBio. 2021 Mar 16;12(2):e00307-21. doi: 10.1128/mBio.00307-21 (PMC8092235; doi:10.1128/mBio.00307-21)
Supplement: Table S2 [file mBio.00307-21-st002.docx]

**Table S2. Primers used in this study**

| Name | Sequence (5’-3’) | Applications |
| --- | --- | --- |
| hph/F | CGGAGACAGAAGATGATATTGAAGGAGC | HYG cassette |
| hph/R | GTTGGAGATTTCAGTAACGTTAAGTGGAT | HYG cassette |
| HF1 | CATCTGCTGCTTGGTGCACGAT | PCR confirm mutant |
| HR2 | TCCGGATCGGACGATTGCGTCG | PCR confirm mutant |
| HR3 | ATAGGTAAGTCAGATTGAATCT | PCR confirm mutant |
| HF4 | GTGGTTGGCTTGTATGGAGCAG | PCR confirm mutant |
| 5915KO/1F | GAATGTGAAGGAAGGAGCCGACTA | Δ*rfw-1* mutant |
| 5915KO/1R | GCTCCTTCAATATCATCTTCTGTCTCCGGGCCTGGGATATTATTGAGGCATG | Δ*rfw-1* mutant |
| 5915KO/2F | ATCCACTTAACGTTACTGAAATCTCCAACGCATGGGAATGTGATATGACGGGT | Δ*rfw-1* mutant |
| 5915KO/2R | GGAGTGACGATAGATACAGCCTGT | Δ*rfw-1* mutant |
| 15snF | GTCAAGGAATGCAACCACCACA | PCR confirm Δ*rfw-1* |
| 15snR | CCACCCACGTATTTGACAACTCC | PCR confirm Δ*rfw-1* |
| 15CF | GAGATAGAGACTGAGGACGACCAG | PCR confirm Δ*rfw-1* |
| 15CR | TAACGTAAGGCTGCACGATGTATC | PCR confirm Δ*rfw-1* |
| 5916KO/F1 | GGAAGGAAGATTAGCAGCAGAAGG | Δ*gt69-2* mutant |
| 5916KO/R1 | GCTCCTTCAATATCATCTTCTGTCTCCGCTGCCTACTGTAGCGTCTGTAC | Δ*gt69-2* mutant |
| 5916KO/F2 | ATCCACTTAACGTTACTGAAATCTCCAACGGGTATGAGCCAGATATGGTTGAG | Δ*gt69-2* mutant |
| 5916KO/R2 | CGAGGAAGAAGATGAACTGGCAGT | Δ*gt69-2* mutant |
| 16snF | GACACAGGTTCCTGTATCCGCT | PCR confirm Δ*gt69-2* |
| 16snR | GTTGACTGTCGGTACAACAGCG | PCR confirm Δ*gt69-2* |
| 16CF | GCCGTTACATTCGCAGCGAAAG | PCR confirm Δ*gt69-2* |
| 16CR | CATGATGTGGTTGGGGTTTCGTTC | PCR confirm Δ*gt69-2* |
| 1516KO/1F | CGAGGAAGAAGATGAACTGGCAGT | Δ*rfw-1*Δ*gt69-2* mutant |
| 1516KO/1R | GCTCCTTCAATATCATCTTCTGTCTCCGTGGTTGAGAATGGCACTACAGCAG | Δ*rfw-1*Δ*gt69-2* mutant |
| 1516CF | CTACGGCAAGATCAGCGAGTTT | PCR confirm Δ*rfw-1*Δ*gt69-2* |
| NAT1-F | TCTCCTGCTTTGCCCGGTGTATGAAAC | NAT1 cassette |
| NAT1-R | GCTTGCAAATTAAAGCCTTCGAGCGTCC | NAT1 cassette |
| NF1 | CGTTGCGTCAGTCCAACATTTG | PCR confirm ΔNCU05915Δ*gt69-2* |
| NR2 | ACGGATCTCCGAGGCCTCGGA | PCR confirm ΔNCU05915Δ*gt69-2* |
| NR3 | TATGGATGTAGCAAAGTACTTA | PCR confirm ΔNCU05915Δ*gt69-2* |
| NF4 | GCTCTACATGAGCATGCCCTGC | PCR confirm ΔNCU05915Δ*gt69-2* |
| 5915NATko/1R | GTTTCATACACCGGGCAAAGCAGGAGAGGGCCTGGGATATTATTGAGGCATG | ΔNCU05915Δ*gt69-2* mutant |
| 5915NATko/2F | GGACGCTCGAAGGCTTTAATTTGCAAGCGCATGGGAATGTGATATGACGGGT | ΔNCU05915Δ*gt69-2* mutant |
| pNLY7/FWD3 | CCACATCGAATTTACCATCCTTTGTTTGAATTCGCGACTTTACCAACAGTCGTTTT | Clone P*ccg1-gfp-gt69-2* to pMF272 |
| pNLY7/REV1 | GCAAGGCAAAGAACACCTCTCGACGCATATTTGGTTGATGTGAGGGGTTGTGAAAG | Clone P*ccg1-gfp-gt69-2* to pMF272 |
| pNLY7/FWD2 | CTTTCACAACCCCTCACATCAACCAAATATGCGTCGAGAGGTGTTCTTTGCCTTGC | Clone P*ccg1-gfp-gt69-2* to pMF272 |
| pNLY6/REV2 | CGGAACCTCTAGAAGCGGCCGCGAATTCGAGATAGAGACTGAGGACGACCAGCAGC | Clone P*ccg1-gfp-gt69-2* to pMF272 |
| pNLY6/FWD3 | GCTGCTGGTCGTCCTCAGTCTCTATCTCGAATTCGCGGCCGCTTCTAGAGGTTCCG | Clone P*ccg1-gfp-gt69-2* to pMF272 |
| pNLY6/REV3 | GCGAGATCTGGGAAAGCTGGTCGTGGCCCTTGTACAGCTCGTCCATGCCGAGAGTG | Clone P*ccg1-gfp-gt69-2* to pMF272 |
| pNLY6/FWD4 | CACTCTCGGCATGGACGAGCTGTACAAGGGCCACGACCAGCTTTCCCAGATCTCGC | Clone P*ccg1-gfp-gt69-2* to pMF272 |
| pNLY7/REV2 | AAAACGACTGTTGGTAAAGTCGCGAATTCAAACAAAGGATGGTAAATTCGATGTGGTCTG | Clone *Pccg1-gfp-gt69-2* to pMF272 |
| pNLY9/FWD3 | GCATCTGAAGTTATTCCACCGCGAGCGCAATTCGCGACTTTACCAACAGTCGTTTTCCA | Clone P*ccg1-gfp-rfw-1* to pMF272 |
| pNLY9/REV1 | GCAGCAAGGAAAAGATAAACGTCAACATATTTGGTTGATGTGAGGGGTTGTGAAAG | Clone P*ccg1-gfp-rfw-1* to pMF272 |
| pNLY9/FWD2 | CTTTCACAACCCCTCACATCAACCAAATATGTTGACGTTTATCTTTTCCTTGCTGCTCC | Clone P*ccg1-gfp-rfw-1* to pMF272 |
| pNLY8/REV2 | CGGAACCTCTAGAAGCGGCCGCGAATTCGAGGTCGATCCGGCAGTCATAGCCAGGA | Clone P*ccg1-gfp-rfw-1* to pMF272 |
| pNLY8/FWD3 | TCCTGGCTATGACTGCCGGATCGACCTCGAATTCGCGGCCGCTTCTAGAGGTTCCG | Clone P*ccg1-gfp-rfw-1* to pMF272 |
| pNLY8/REV3 | TTGGGCAGAAGCCCCAGAGCCTTGCGCCCTTGTACAGCTCGTCCATGCCGAGAGTG | Clone P*ccg1-gfp-rfw-1* to pMF272 |
| pNLY8/FWD4 | CACTCTCGGCATGGACGAGCTGTACAAGGGCGCAAGGCTCTGGGGCTTCTGCCCAA | Clone P*ccg1-gfp-rfw-1* to pMF272 |
| pNLY9/FWD2 | CTTTCACAACCCCTCACATCAACCAAATATGTTGACGTTTATCTTTTCCTTGCTGCTCC | Clone P*ccg1-gfp-rfw-1* to pMF272 |
| pNLY11/FWD3 | GTACTCAAACTCAACTAGTGTCTATTTCAATTCGCGACTTTACCAACAGTCGTTTT | Clone P*ccg1-gfp-gt69-2^JW224^* to pMF272 |
| pNLY11/REV1 | GCCAAGCAAAGAGAACTGCCCGACTCATATTTGGTTGATGTGAGGGGTTGTGAAAG | Clone P*ccg1-gfp-gt69-2^JW224^* to pMF272 |
| pNLY11/FWD2 | CTTTCACAACCCCTCACATCAACCAAATATGAGTCGGGCAGTTCTCTTTGCTTGGC | Clone P*ccg1-gfp-gt69-2^JW224^* to pMF272 |
| pNLY10/REV2 | CGGAACCTCTAGAAGCGGCCGCGAATTCGGATAGCTGGTCATGGCCGAGATACCAG | Clone P*ccg1-gfp-gt69-2^JW224^* to pMF272 |
| pNLY10/FWD3 | CTGGTATCTCGGCCATGACCAGCTATCCGAATTCGCGGCCGCTTCTAGAGGTTCCG | Clone P*ccg1-gfp-gt69-2^JW224^* to pMF272 |
| pNLY10/REV3 | GAAAGGATGATGGCGGTGTGTGCACGTTCTTGTACAGCTCGTCCATGCCGAGAGTG | Clone P*ccg1-gfp-gt69-2^JW224^* to pMF272 |
| pNLY10/FWD4 | CACTCTCGGCATGGACGAGCTGTACAAGAACGTGCACACACCGCCATCATCCTTTC | Clone P*ccg1-gfp-gt69-2^JW224^* to pMF272 |
| pNLY10/REV4 | AAAACGACTGTTGGTAAAGTCGCGAATTGAAATAGACACTAGTTGAGTTTGAGTACTGGTAGGA | Clone P*ccg1-gfp-gt69-2^JW224^* to pMF272 |
